# Exosomal circTGFBR2 promotes hepatocellular carcinoma progression via enhancing ATG5 mediated protective autophagy

**DOI:** 10.1038/s41419-023-05989-5

**Published:** 2023-07-20

**Authors:** Xin Wang, Feng-Lin Dong, Ying-Qiao Wang, Hong-Long Wei, Tao Li, Jie Li

**Affiliations:** 1 Shandong First Medical University & Shandong Academy of Medical Sciences, Jinan, 250117 China; 2 Department of General Surgery, The First Affiliated Hospital of Shandong First Medical University & Shandong Provincial Qianfoshan Hospital, Jinan, 250014 China; 3 Department of Hematology, The Third Affiliated Hospital of Shandong First Medical University, Jinan, 250014 China

**Keywords:** Liver cancer, Non-coding RNAs

## Abstract

Exosomes contribute substantially to the communication between tumor cells and normal cells. Benefiting from the stable structure, circular RNAs (circRNAs) are believed to serve an important function in exosome-mediated intercellular communication. Here, we focused on circRNAs enriched in starvation-stressed hepatocytic exosomes and further investigated their function and mechanism in hepatocellular carcinoma (HCC) progression. Differentially expressed circRNAs in exosomes were identified by RNA sequencing, and circTGFBR2 was identified and chosen for further study. The molecular mechanism of circTGFBR2 in HCC was demonstrated by RNA pulldown, RIP, dual-luciferase reporter assays, rescue experiments and tumor xenograft assay both in vitro and vivo. We confirmed exosomes with enriched circTGFBR2 led to an upregulated resistance of HCC cells to starvation stress. Mechanistically, circTGFBR2 delivered into HCC cells via exosomes serves as a competing endogenous RNA by binding miR-205-5p to facilitate ATG5 expression and enhance autophagy in HCC cells, resulting in resistance to starvation. Thus, we revealed that circTGFBR2 is a novel tumor promoter circRNA in hepatocytic exosomes and promotes HCC progression by enhancing ATG5–mediated protective autophagy via the circTGFBR2/miR-205-5p/ATG5 axis, which may be a potential therapeutic target for HCC.

## Background

Hepatocellular carcinoma (HCC) is among the top 5 causes of cancer-related death in 90 countries worldwide, and the number of new cases per year is predicted to increase by 55.0% between 2020 and 2040 [1]. Rapid advances in diagnostic and therapeutic techniques have effectively improved the prognosis of HCC patients in the early or intermediate stages [2]. However, effective control of tumor progression for patients at an advanced stage remains challenging. As a highly conserved cellular degradation system, autophagy is considered to play an important role in HCC progression, especially in response to environmental stressors faced by tumor cells, such as starvation, hypoxia, growth factor deprivation, and antivascular therapy [3]. Notably, evidence now suggests that autophagy in both tumor cells and the host as well as the surrounding tumor microenvironment (TME) can promote tumor adaptation, growth and progression, and resistance to therapy [4, 5]. Therefore, understanding the crosstalk between tumor cell autophagy and the TME in HCC progression, especially the initiation and regulation of protective autophagy, which contributes a lot to malignant progression and therapeutic escape, is beneficial for developing more effective strategies for HCC treatment and improving patient outcomes.

Exosomes are a subgroup of extracellular vesicles (EVs) with a 30-150 nm diameter that are released from living cells and can be transported to nearby or distant cells to deliver cellular molecular components, including proteins, DNA, messenger RNAs (mRNAs), microRNAs (miRNAs), circular RNAs (circRNAs) and other noncoding RNAs, to facilitate intercellular communication [6, 7]. Studies have confirmed that exosomes contribute substantially to the communication between tumor cells and normal cells and are involved in a variety of cancer-related biological processes, such as cell proliferation, apoptosis, and therapeutic resistance [7, 8]. Recently, exosome-mediated transfer of circRNAs has also been identified as a novel mechanism for the regulation of tumor progression and has attracted widespread attention [9–11].

CircRNAs are a class of noncoding RNAs that form a closed continuous loop through covalent junctions at the 3′ and 5′ ends and have a stable, covalent molecular structure that is resistant to degradation by RNase R [12, 13]. Recently, with the development of high-throughput sequencing and bioinformatics analysis technologies, circRNAs have emerged as a research hotspot in the biomedical field. Evidence shows that circRNAs are abundantly and specifically expressed in both tumor and normal tissues, and circRNAs are also highly enriched and stable in exosomes [14, 15]. Generally, circRNAs are thought to act as endogenous sponges to adsorb miRNAs, while miRNAs silence target genes post-transcriptionally by binding to the 3′-untranslated region (3′UTR) of mRNAs [16, 17]. In addition, circRNAs can exert their biological activity through interaction with proteins or by directly encoding proteins [18, 19]. In HCC, circRNAs have been reported to be involved in a variety of tumor-malignant behaviors. However, the majority of circRNAs and their functions are still not fully elucidated. Further studies are required to confirm the role of circRNAs in this intercellular communication and the related mechanisms.

In the present study, we observed that exosomes derived from starvation-stressed THLE-2 cells (Exo-Ts) could enhance protective autophagy in the HCC cell lines Hep3B and Huh-7, and therefore we focused specifically on the effect of exosomal contents on the progression of hepatocellular carcinoma and investigated the regulatory targets and mechanisms of exosomal contents to promote HCC progression. For this, we further performed high-throughput sequencing of THLE-2 cells and their exosomes and found that circTGFBR2 (circBase ID: hsa_circ_0005224) was significantly overexpressed in Exo-Ts. We further confirmed that circTGFBR2 was the major factor in Exo-Ts for enhancing protective autophagy in HCC cells to resist starvation stress through in vitro and in vivo experiments. Mechanistically, upon delivery into HCC cells by exosomes, circTGFBR2 could elevate autophagy-related protein 5 (ATG5) expression by sponging and inducing degradation of miR-205-5p, thereby enhancing ATG5-mediated protective autophagy and promoting progression of HCC cells. The circTGFBR2/miR-205-5p/ATG5 axis might serve as a promising therapeutic target in HCC treatment.

## Materials and methods

### Cell lines

HCC cells (Hep3B and Huh7) were purchased from the Typical Culture Reserve Center of China (Shanghai, China), and human hepatocytes (THLE-2) were purchased from Cellcook Biotech Company (Guangzhou, China). All cell lines were recently authenticated by short tandem repeat DNA profiling and excluded for mycoplasma infection . Hep3B and Huh-7 cells were cultured in DMEM (Gibco, Carlsbad, NY, USA), while THLE-2 cells were cultured in BEGM (Gibco). Additionally, 10% fetal bovine serum (Gibco) and 1% penicillin‒streptomycin solution (Gibco) were added to the medium. Earle’s balanced salt solution (EBSS, Gibco) was used as a substitute for complete medium to establish a starvation-stressed model for cells. To establish stable circTGFBR2-overexpressing Hep3B cells (Hep3B oe-ciR), we transfected the cells with pSLenti-EF1-EGFP-F2A-Puro-CMV-S-circTGFBR2-WPRE vectors containing front and rear circular frames, which were designed and produced by OBiO Technology (Shanghai, China). The control group for overexpression was transfected with the EGFP vector in Hep3B cells (Hep3B vector). The efficiency and stability of overexpression were verified by qRT‒PCR using different circTGFBR2 primers.

### Tissue specimens and blood samples

Before sample collection, approval was obtained from the Medical Ethics Committee of The First Hospital Affiliated with Shandong First Medical University and informed consent was obtained from all subjects. A total of 20 HCC tissues and matched peritumoral liver tissues were obtained from The First Hospital Affiliated with Shandong First Medical University (Shandong Province, China) between 2020 and 2022 (10 patients that did not receive any antitumor treatment before surgery and 10 patients that received only transcatheter arterial embolization (TAE) treatment before surgery). All tissues were stored at −80 °C before RNA and protein extraction. A total of 19 blood samples from patients with pathologically confirmed HCC were obtained from The First Hospital Affiliated with Shandong First Medical University (Shandong Province, China), including 12 who had received TAE treatment and 7 who had not. Meanwhile, 10 blood samples from healthy adults were collected as negative controls. All blood samples were collected using EDTA-containing tubes and the circulating exosomes were separated by ultracentrifugation.

### Isolation of exosomes

THLE-2 cells were cultured in BEGM with 10% exosome-depleted FBS. After collection, the medium was sequentially centrifuged in a 4 °C environment at 300 × g for 10 min to remove the cell pellet, 2000 × g for 10 min to remove the dead cells, and 10,000 × g for 10 min at 4 °C to remove the cell debris. Finally, the supernatant was centrifuged at 10,0000 g for 90 min at 4 °C, and the exosome precipitate was resuspended in precooled phosphate-buffered saline (PBS). Nanoparticle tracking analysis (NTA, NanoSight NS300, Malvern, UK), transmission electron microscopy (TEM, G2 spititi FEI, Tecnai, USA), and detection of marker proteins (CD9, CD63, CD81, LAMP2 and TSG101) were used to identify exosomes. An exosome standard (HEK293 cell line, Novus, USA) was analyzed as a positive control, and THLE-2 cell lysate was performed as a negative control. The exosomal RNA Isolation Kit (Norgen, CA) was used to extract total RNA from exosomes for further analysis.

### Exosome labeling and tracking

A green dye PKH67 kit (MINI67-1KT, Sigma, USA) was used to label the isolated exosomes according to the manufacturer’s instructions. The labeled exosomes were cocultured with HCC cells and incubated for 24 h. Cells were observed and photographed using a fluorescence microscope (Olympus FSX100, Tokyo, Japan).

### RNA extraction and PCR assay

Total RNA was isolated from the tissues and cell lines using TRIzol reagent (Invitrogen, Waltham, MA, USA), while miRNA was isolated by the miRcute miRNA Isolation Kit (Tiangen Biotech, Beijing, China) according to the instructions of the manufacturer. Complementary DNA was synthesized using random primers and the FastKing RT Kit for circRNA and mRNA (Tiangen Biotech, Beijing, China) or the miRcute Plus miRNA First-Strand cDNA Kit for miRNA (A-tailing method, Tiangen Biotech, Beijing, China). Quantitative real-time PCR (qRT‒PCR) assays were performed using the SYBR Green SuperReal PreMix Plus Kit (Tiangen Biotech, Beijing, China) or miRcute Plus miRNA qPCR Kit (Tiangen Biotech, Beijing, China) on a real-time fluorescence quantitative PCR system (CFX96, Bio-Rad, Hercules, CA). The differences between the circRNA and miRNA were normalized to GAPDH or U6 levels. The primer details are listed in Supplementary Table 1.

### RNA sequencing (RNA-seq) analysis

An exosomal RNA isolation kit (Norgen Biotek, CA) was used to extract total RNA from exosomes. The extracted RNA was quantified using a NanoDrop ND-2000 (Thermo Scientific) and detected by an Agilent Bioanalyzer 2100 (Agilent Technologies) for RNA integrity. Total RNA was purified using the Qiagen RNeasy Kit and then taken for amplification and labeling (using Cyanine-3-CTP (Cy3) dye). The labeled cDNA was finally hybridized with an oligo microarray at 65 °C for 17 h. The original images were scanned using Agilent Scanner G5761A (Agilent Technologies) after elution, and the raw data were extracted using Feature Extraction software (version 12.0.3.1, Agilent Technologies). Genespring software (version 14.8, Agilent Technologies) was used for quantile normalization and subsequent processing. The normalized data were filtered, and differentially expressed genes were screened using a *t*-test with the criteria of *P-*value < 0.05 and absolute value of log2(fold change) (log2FC) > 1.

### Cell transfection

The overexpression vectors for circ_0005224 (oe-ciR) and ATG5 (oe-ATG5) were designed and synthesized by OBiO Technology (Shanghai, China). Circ_0005224 was cloned into a pSLenti-EF1-F2A-Puro-CMV-S-circRNA-WPRE vector containing front and rear circular frames, while the ATG5 CDS or 3′UTR was cloned into a pSLenti-CMV-MCS-3xFLAG-PGK-Puro-WPRE vector. The small interfering RNAs against circ_0005224 (si-ciR) or other candidate circRNAs and negative control (NC) were designed and produced by GenePharma (Shanghai, China), as well as the mimics and inhibitor of miR-205-5p. For higher transfection efficiency, we applied the jetPRIME reagent (Polyplus Transfection, Illkirch, FRANCE) when cells in the 6-well plates reached 70% confluence. qRT‒PCR was conducted to validate the transfection efficiency of the above vectors. The detailed sequences of the small interfering RNAs are listed in Supplementary Table 2.

### Western blotting

Western blotting was conducted to detect the protein levels of CD9 (1:1000, Abcam, Ab92726), CD63 (1:1000, Abcam, Ab216130), TSG101 (1:1000, Abcam, Ab125011), Calnexin (1:2000, Abcam), CD81 (1:500, Abcam, Ab79559), LAMP2 (1:2000, Abcam, Ab199946), LC3B (1:1000, Cell Signaling Technology, #43566), p62 (1:1000, Cell Signaling Technology, #88588), ATG5 (1:1000, Cell Signaling Technology, #9980) and GAPDH (1:1000, Proteintech, 10494-1-AP) according to our previous reports [20, 21].

### Observation of autophagic flux

Ad-mCherry-GFP-LC3 (Beyotime, Shanghai, China) was transferred to HCC cells in different treatment groups. Cells were observed and photographed using a fluorescence microscope (DMi8, Leica, German) after 24 h of incubation. Moreover, autophagic vacuoles were observed and identified by TEM according to guidelines for the use and interpretation of assays for monitoring autophagy (4th edition) [22].

### Apoptosis detection

The apoptotic rate of HCC cells was detected by Annexin V-FITC/PI Apoptosis Detection Kits (Dojindo, Japan). Briefly, after coculture with Exo-Ts or introduction of RNA fragments for 48 h, HCC cells were cultured in EBSS or full DMEM for 12 h. Then, cells (5 × 10^5^) were trypsinized by EDTA-free trypsin and washed twice with cold PBS. One microliter of annexin V-FITC and 1 μL of PI working solution were used to stain cells in 100 μL of binding buffer for 15 min at room temperature in the dark. The apoptosis index was determined using a FACSCalibur flow cytometer (Becton Dickinson).

### Ribonuclease R (RNase R) resistance assay and actinomycin D (ActD) assay

RNase R (2.5 U/μg, Geneseed, Guangzhou, China) resistance assays were implemented for the identification of circRNA according to our previous reports [19], while the qRT‒PCR assay was used to analyze the relative expression of circTGFBR2, TGFBR2, and GAPDH compared to the control group. An actinomycin D (ActD) assay (MedChemExpress, New Jersey, USA) was used to detect the stability of RNA. Starved THLE-2 cells were treated with 1 μg/ml ActD reagent. Total RNA was extracted after 0, 6, 12, 18, or 24 h of administration for further qRT‒PCR assays.

### RNA immunoprecipitation (RIP) assay

The RIP assay was performed using the EZMagna RIP kit (Millipore, MA, USA). Briefly, Hep3B and Huh-7 cells were lysed in RIP lysis buffer, followed by incubation with RIP buffer containing magnetic beads conjugated with anti-AGO2 antibody (Millipore) or anti-EIF4A3 antibody (Millipore). Normal mouse IgG (Millipore) was used as a negative control. Immunoprecipitated proteins were digested with proteinase K, and the associated RNA was purified and analyzed by qRT‒PCR using divergent primers for circTGFBR2.

### RNA pulldown assay with biotin-labeled circTGFBR2 probe

The biotin-labeled circTGFBR2 probe complementary to the backspliced junction and the negative control (NC) probe were synthesized by OBiO Technology (Shanghai, China), and the sequences of the biotin-labeled RNA probes are listed in Supplementary Table 3. CircTGFBR2-overexpressing Hep3B and Huh-7 cells were lysed with lysis buffer and incubated with biotin-labeled circTGFBR2 probes at 4 °C overnight in RNA‒RNA hybridization buffer. Cell lysates were then incubated with streptavidin-coated magnetic beads for 4 h at room temperature and washed in wash buffer. After washing, the RNA complexes bound to the beads were eluted and extracted by the miRcute miRNA Isolation Kit (Tiangen Biotech) for qRT‒PCR.

### RNA pulldown assay with biotin-labeled miRNA

The biotin-labeled miRNA mimics or mutants were synthesized by OBiO Technology (Shanghai, China), and the sequences of the biotin-labeled RNA oligos are listed in Supplementary Table 3. In brief, circTGFBR2-overexpressing Hep3B and Huh-7 cells were transfected with biotin-labeled miR-205-5p mimics or mutants and collected 48 h after transfection. The cells were lysed with lysis buffer, and 50 μl of the cell lysates were aliquoted for input. The remaining cell lysates were incubated with streptavidin-coated magnetic beads at 4 °C for 4 h at room temperature and then washed in wash buffer. After washing, the RNA complexes bound to the beads were eluted and extracted by an RNA Clean Kit (Tiangen Biotech) for qRT‒PCR.

### Fluorescence in situ hybridization (FISH) assay

Hep3B and Huh-7 cells were seeded in dishes and cocultured with Exo-Ts in exosome-depleted medium. After 48 h, the cells were fixed at room temperature with 4% paraformaldehyde and treated with protease K (20 µg/ml). Then, the cells were overlaid with a Cy3-labeled circTGFBR2 probe and FAM-labeled miR-205-5p probe (Servicebio, Wuhan, China) at 37 °C for 48 h. The signals of the probe were detected by a confocal microscope (Nikon A1 + , Minato, Japan). Nuclei were counterstained with DAPI.

### Dual-luciferase reporter assay

Firefly/Renilla luciferase vectors (pMIR-REPORT, OBiO Technology) bearing the sequence of circTGFBR2 or the 3′UTR of ATG5 mRNA (wild type or mutant) were introduced into 293 T cells along with miR-205-5p mimics. After 48 h, firefly and Renilla luciferase activities were measured using a Dual-Luciferase Reporter Assay System (E1960, Promega) according to the manufacturer’s protocol.

### Tumor xenograft assay

Before the experiment, we obtained consent from the Institutional Animal Care and Use Committee of The First Hospital Affiliated with Shandong First Medical University. BALB/c nude mice (female, 5–6 weeks, 18–22 g) were purchased from GemPharmatech Co., Ltd. (Certificate number: SCXK 2018-0008, Jiangsu, China). All animals were kept in a pathogen-free environment and fed ad libitum. Twelve mice were randomly (random number grouping method) divided into two groups: the “vector” group (negative control) and the “oe-circTGFBR2” group (overexpressed crcTGFBR2). A total of 5 × 10^6^ cells (Hep3B (vector) or Hep3B (oe-ciR)) suspended in 200 μl of PBS were subcutaneously injected into the right flank of mice. Tumor volume was measured and recorded every 2 days using the following formula: volume (mm^3^) = length (mm) × width^2^ (mm^2^)/2. After 26 days, the mice were sacrificed, and the tumors were weighed. Next, fifteen mice were randomly (random number grouping method) divided into three groups: the “Exo-T” group (exosomes divided from normal THLE-2 cells), the “Exo-Ts” group (exosomes divided from starvation-stressed THLE-2 cells) and the “Exo-Ts ^si-ciR^” group (exosomes divided from starvation-stressed circTGFBR2 knockdown THLE-2 cells). The mice received alternate-day intraperitoneal injections (i.p) of exosomes divided from the accordingly treated THLE-2 cells at a dose of 10 mg/kg. Tumor volume was measured and recorded every 2 days. After 20 days, the mice were sacrificed, and the tumors were weighed.

### Immunohistochemistry (IHC)

IHC assays were performed on xenograft tumor tissues using antibodies against Ki‐67 (1:100, Abcam) and ATG5 (1:100, Abcam) as described in our previous studies [20, 23]. The staining results were quantified using Image-Pro Plus 6.0 (Media Cybernetics, USA) and are shown as the percentage of positive cells (for Ki-67) or the mean density (IOD sum/area, for ATG5).

### TdT-mediated dUTP Nick-End Labeling (TUNEL)

TUNEL assays were performed according to the manufacturer’s instructions of the CF488 (Green) TUNEL Cell Apoptosis Detection Kit (Servicebio, Wuhan, China) to detect cell apoptosis in xenograft tumor tissues. The assay was stained in the green channel at 515 nm, while DAPI was applied as a nuclear counterstain in the blue channel at 461 nm. Images were taken with a fluorescence microscope (Leica, German), and the results were quantified using Image-Pro Plus 6.0 and are shown as the percentage of positive cells.

### Statistical analysis

GraphPad Prism 9 (GraphPad Software, LLC) was used to quantify and analyze the data, and the data are presented as the mean ± standard deviation. Student’s *t*-test, one‐way analysis of variance (ANOVA), two‐way ANOVA, and χ2 test were used to analyze the differences between groups. The correlation between circTGFBR2 and miR-205-5p or the correlation between miR-205-5p and ATG5 mRNA in human tissues was determined with linear regression analysis. *P* < 0.05 was considered statistically significant.

## Results

### Exosomes derived from starvation-stressed THLE-2 cells enhance the protective autophagy of HCC cells in vitro

**Fig. 1:**
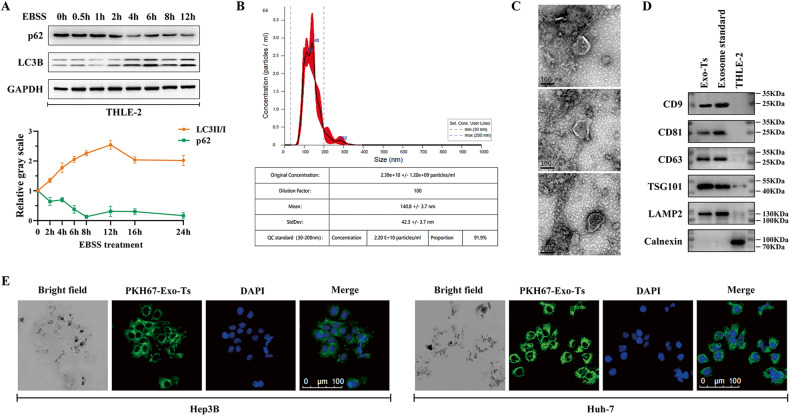
Characterization of exosomes derived from starvation-stressed THLE-2 cells. **A** EBSS was used to establish a starvation-stressed model of THLE-2 cells, and the autophagic intensity of cells was evaluated by detecting the autophagy-related proteins LC3B and p62. The data are presented as the mean ± SD of at least three independent experiments. **B** The size range of the exosomes isolated from starvation-stressed THLE-2 cells (Exo-Ts) identified by NAT analysis. **C** TEM image of Exo-Ts. **D** Western blotting analysis of exosomal markers, including CD9, CD63, CD81, LAMP2, TSG101 and Calnexin. An exosome standard from HEK293 cell line was analyzed as a positive control while THLE-2 cell lysate was as a negative control. **E** The fluorescent signal of PKH67 labeled Exo-Ts was captured in HCC cell lines.

To explore the mechanism of exosome-based regulation of autophagy in HCC, we established a starvation-stressed model of THLE-2 cells using EBSS and performed western blotting assays to evaluate the autophagic intensity of cells by detecting the autophagy-related proteins LC3B and p62 (Fig. 1A). According to the results above, THLE-2 cells were treated with EBSS or cultured in BEGM with 10% exosome-depleted FBS for 12 h. Then, exosomes secreted by starvation-stressed THLE-2 cells (Exo-Ts) and normal THLE-2 cells (Exo-T) were isolated and identified. The NTA results showed that the average particle size in the extracted exosome samples was 140.8 ± 3.7, and the percentage of particles in the distribution range of 30–200 nm was 91.9% (Fig. 1B). Cup-shaped nanoparticles with a bilayer membrane structure were visible in the sample under TEM views, which was characteristic of exosomes [24] (Fig. 1C). Western blotting analysis confirmed the presence of CD9, CD63, CD81, LAMP2 and TSG101 as well as the absence of calnexin in the extracted exosome samples, which were reported as markers of exosomes [25] (Fig. 1D). The above results suggest that exosomes (Exo-Ts and Exo-T) were successfully isolated from THLE-2 cell lines. The isolated exosomes were resuspended in PBS and cocultured with Hep3B and Huh-7 cells after labeling with PKH67. After 24 h, the fluorescent signal of PKH67 was captured in both Hep3B and Huh-7 cells by confocal microscopy, suggesting that HCC cells were taking in exosomes (Fig. 1E).

**Fig. 2:**
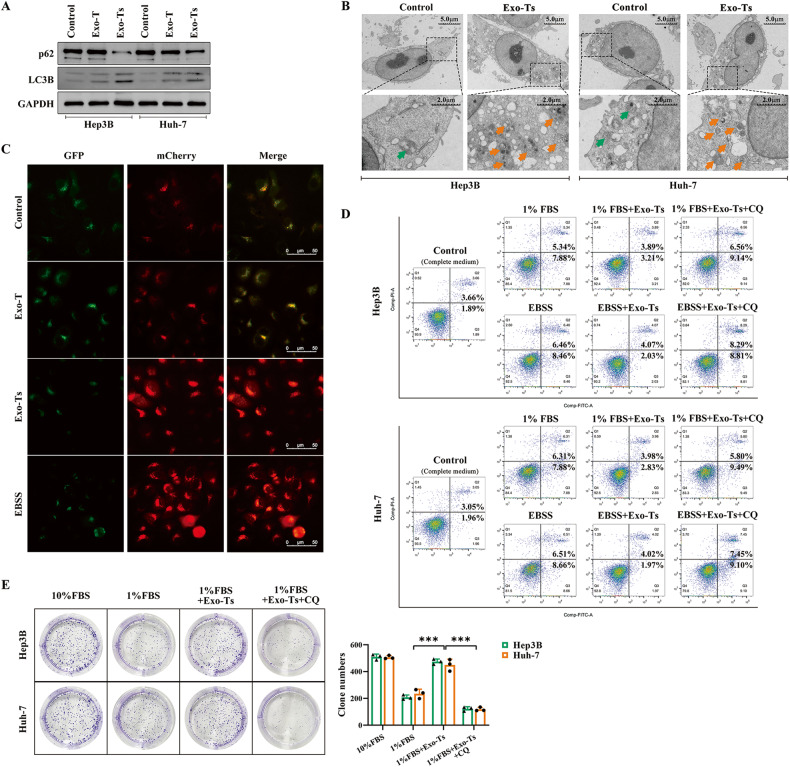
Exo-Ts enhance the protective autophagy of HCC cells in vitro. **A** Western blotting analysis of LC3B and p62 in Hep3B and Huh-7 cells after cocultured with exosomes derived from normal THLE-2 cells (Exo-T) or starvation-stressed THLE-2 cells (Exo-Ts). **B** Visualization of autophagic compartments via TEM. Arrows indicate double-membraned vacuoles. **C** Microscopic observation of autophagic flux after transfection of Ad-mCherry-GFP-LC3 for 24 h. The red puncta represent autolysosomes and the yellow puncta (merge of both green and red signals) represent autophagosomes. The autophagosomal and autolysosomal abundance is measured by the number of puncta: a higher density of yellow and red puncta indicates a higher level of autophagic flux. **D** The apoptotic rate of HCC cells was determined by Annexin V/PI staining using flow cytometry analysis after the indicated treatment. **E** The proliferation of HCC cells in low serum concentrations was assessed by colony formation assays after coculture with Exo-Ts alone or in combination with chloroquine (CQ). The data are presented as the mean ± SD of at least three independent experiments. ***P < 0.001.

Considering that exosomes have activities as diverse as transmitting signals and molecules to other cells [26], we specifically focused on the variations in autophagy in HCC cells after ingesting Exo-Ts. The western blotting assay showed that coculturing with Exo-Ts could significantly increase LC3B lipidation and decrease p62 accumulation in Hep3B and Huh-7 cells, suggesting that exosomes from starved THLE-2 cells were capable of enhancing autophagy in their target HCC cells (Fig. 2A). TEM observations showed that Exo-Ts significantly increased the number of double-membraned vacuoles to a relatively high level in HCC cells, suggesting that cellular autophagy was activated (Fig. 2B). Moreover, autophagic flux in HCC cells was observed after Ad-mCherry-GFP-LC3 adenovirus transfection according to the amount of yellow (the merge of mCherry and GFP signal, autophagosome) and red (mCherry signal, autolysosome) puncta in cells. Compared to Exo-T, Exo-Ts significantly increased the number of yellow (autophagosome) and red (autolysosome) puncta in Hep3B and Huh-7 cells, demonstrating a facilitative effect on autophagic flux (Fig. 2C). Annexin V PI/FITC was used for cell apoptosis through flow cytometry. The results demonstrated that Exo-Ts significantly reduced apoptosis in Hep3B and Huh-7 cells under starvation stress, while chloroquine (CQ) reversed this phenomenon and significantly increased the rate of apoptosis in HCC cells (Fig. 2D). Additionally, a colony formation assay was performed to evaluate the proliferation of cells in low serum concentrations (1% FBS). The number of colonies formed by the HCC cells cocultured with Exo-Ts was significantly greater than that formed by the cells in the control group, while CQ was able to reverse this effect of Exo-Ts (Fig. 2E). These results, therefore, confirmed that exosomes derived from starved THLE-2 cells could enhance autophagy in HCC cells, which helped target cells resist starvation stress.

### Circular transcript hsa_circ_0005224 is significantly upregulated in Exo-Ts and may be responsible for exosome-enhanced autophagy in HCC cells

**Fig. 3:**
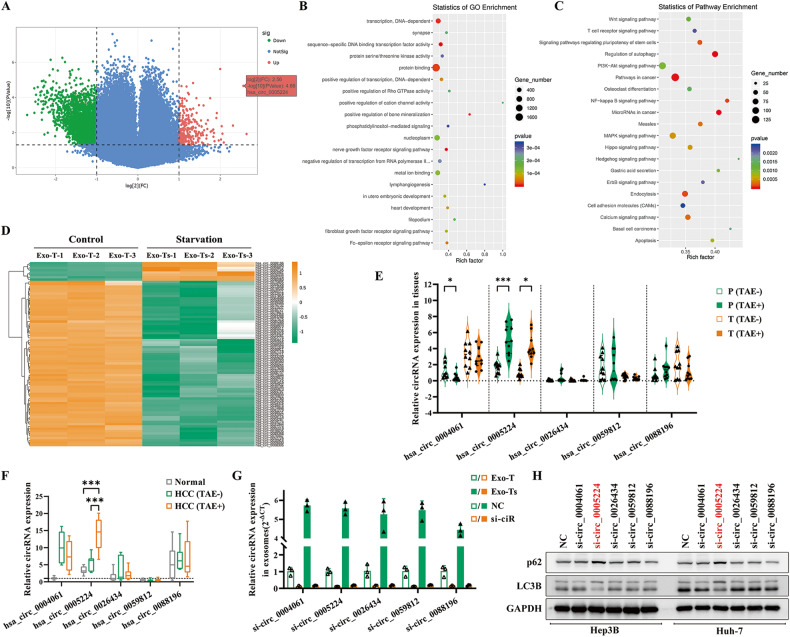
Hsa_circ_0005224 (circTGFBR2) is significantly enriched in Exo-Ts. **A** The differentially expressed circRNAs in Exo-Ts compared to Exo-T were visualized with volcano plots; the log2(fold change) (log2FC) and the −log10(*P*-value) are respectively plotted on the X and Y axes. The dashed lines signify the filtering criteria (*P* < 0.05, fold change ≥ 2). Upregulated circRNAs are shown in red, and downregulated circRNAs are shown in green. **B**, **C** GO and KEGG analyses of the host genes of differentially expressed circRNAs in exosomes. **D** Hierarchical cluster analysis shows the differentially expressed circRNAs at a cutoff criterion of |log2FC | > 2.0 and *P* < 0.05. The columns represent the different exosome samples. Each row indicates a circular transcript, and the colors represent the abundance of the transcripts. **E** The expression of candidate circRNAs in HCC tumor tissues and matched peritumoral liver tissues obtained from patients with or without preoperative TAE was measured by qRT‒PCR assays. **F** qRT-PCR assays were performed to check the content of candidate circRNAs in circulating exosomes isolated from the blood samples. **G** Relative expression changes of candidate circRNAs in Exo-T and Exo-Ts after transfection of the corresponding siRNAs aimed at the splice sequences of target circRNAs (si-ciRs) into THLE-2 cells. **H** Western blotting analysis of LC3B and p62 in Hep3B and Huh-7 cells after cocultured with exosomes derived from si-ciRs treated THLE-2 cells. The data are presented as the mean ± SD of at least three independent experiments. ***P < 0.001, *P < 0.05.

To obtain the expression profiles and identify differentially expressed circRNAs in exosomes, we performed RNA-seq in Exo-Ts and Exo-T. Through RNA-seq of 3 matched samples of Exo-Ts and Exo-T, 86935 circRNAs were detected. The significantly differentially expressed circRNAs between the two groups are presented as volcano plots at a cutoff criterion of |log2FC | > 1.0 and *P* < 0.05, including 257 significantly upregulated and 2617 significantly downregulated circRNAs (Fig. 3A). GO and KEGG analyses of the host genes of differentially expressed circRNAs are shown in Fig. 3B and Fig. 3C. The results of hierarchical clustering at a cutoff criterion of |log2FC | > 2.0 and *P* < 0.05 are displayed in a heatmap (Fig. 3D), and the five circRNAs with the most significant upregulated expression in Exo-Ts were candidates for further study (Table 1). As hypoxia may arise as a result of TAE and lead to the activation of autophagy in local tissues [27, 28], the expression of candidate circRNAs in HCC tumor tissues and matched peritumoral liver tissues obtained from patients with or without preoperative TAE was measured by qRT‒PCR assays. The results demonstrated that the expression of hsa_circ_0005224 in both tumor tissues and peritumoral normal tissues was significantly upregulated in patients who received TAE treatments before surgery compared to those who declined TAE treatments (Fig. 3E). The relationship between the expression of hsa_circ_0005224 in tissue samples and the clinical features of cases is shown in Table 2. Moreover, qRT-PCR assays were performed to check the content of candidate circRNAs in circulating exosomes isolated from the blood samples. The results showed that the circulating exosomes from HCC (TAE+) group contained significantly higher levels of hsa_circ_0005224 (circTGFBR2) than those from normal and HCC (TAE-) groups, suggesting that circTGFBR2 might be transmitted via exosomes in HCC patients especially those had accepted TAE treatment (Fig. 3F). To further validate the role of candidate RNAs in exosome-enhanced autophagy in HCC cells, we designed siRNAs aimed at the splice sequences of target circRNAs and transfected them into THLE-2 cells. Then, exosomes were isolated after 48 h for qRT‒PCR or coculture with HCC cells. The results of qRT‒PCR assays verified that the expression of the corresponding circRNAs in exosomes was significantly downregulated (Fig. 3G). Western blotting assays showed that knockdown of hsa_circ_0005224 in Exo-Ts eliminated its ability to enhance autophagy in HCC cells (Fig. 3H). These above findings indicated that hsa_circ_0005224 might play a key role in the process of exosome-enhanced autophagy in HCC cells.

**Table 1:** Circbase ID and information of the candidate circRNAs.

Circbase ID	Log2 fold charge	*P*-value	Gene symbol	Spliced seq length
hsa_circ_0004061	2.641330315	0.012035034	FMN2	793 bp
hsa_circ_0005224	2.56392336	0.000021789	TGFBR2	360 bp
hsa_circ_0026434	2.311412189	0.003979419	KRT6A	1286 bp
hsa_circ_0059812	2.087989853	0.036785192	BPIFB2	502 bp
hsa_circ_0088196	2.054921614	0.011559476	TNC	1206 bp

**Table 2:** Relationship between expression of hsa_circ_0005224 in tissue samples and clinical features of cases.

Clinical features		Relative expression of hsa_circ_0005224			
Characteristics	*n* = 20	Peritumoral liver tissues	*P*-value	Tumor tissues	*P-*value
**Gender**					
male	13	3.464 ± 2.428	0.888	2.309 ± 1.808	0.497
female	7	3.314 ± 1.838		2.978 ± 2.472	
**Age**					
<60	11	3.763 ± 2.376	0.441	2.609 ± 1.858	0.877
≥60	9	2.981 ± 1.989		2.463 ± 2.326	
**TAE before surgery**					
No	10	1.729 ± 0.794	<0.001^***^	0.903 ± 0.569	<0.001^***^
Yes	10	5.093 ± 1.791		4.184 ± 1.535	
**HbsAg**					
Negative	5	2.310 ± 1.229	0.094	1.256 ± 1.013	0.111
Positive	15	3.778 ± 2.345		2.963 ± 2.126	
**AFP (ng/ml)**					
<400	16	3.777 ± 2.297	0.014^*^	2.872 ± 2.127	0.152
≥400	4	1.949 ± 0.669		1.229 ± 0.686	
**Cirrhosis**					
Negative	8	1.828 ± 0.929	0.002^**^	1.060 ± 0.719	0.004^**^
Positive	12	4.467 ± 2.167		3.532 ± 2.022	
**Child-Pugh stage**					
A	12	2.464 ± 1.391	0.034^*^	2.109 ± 2.020	0.25
B	8	4.832 ± 2.476		3.196 ± 1.806	
**Tumor size**					
<3 cm	7	3.276 ± 1.826	0.846	2.638 ± 2.316	0.883
≥3 cm	13	3.484 ± 2.431		2.492 ± 1.951	
**MVI**					
No	9	3.884 ± 2.017	0.397	2.981 ± 2.156	0.397
Yes	11	3.024 ± 2.344		2.186 ± 1.939	
**ES nuclear grade**					
I ~ II	12	3.929 ± 2.132	0.204	2.917 ± 2.194	0.325
III ~ IV	8	2.635 ± 2.175		1.983 ± 1.723	

*P < 0.05, **P < 0.01, ***P < 0.001;

*TAE* transcatheter arterial embolization, *AFP* alpha fetoprotein, *MVI* microvascular invasion, *ES nuclear grade* Edmondson-Steiner nuclear grade.

### Hsa_circ_0005224 (circTGFBR2) is the key effector cargo in Exo-Ts to enhance protective autophagy in HCC cells

**Fig. 4:**
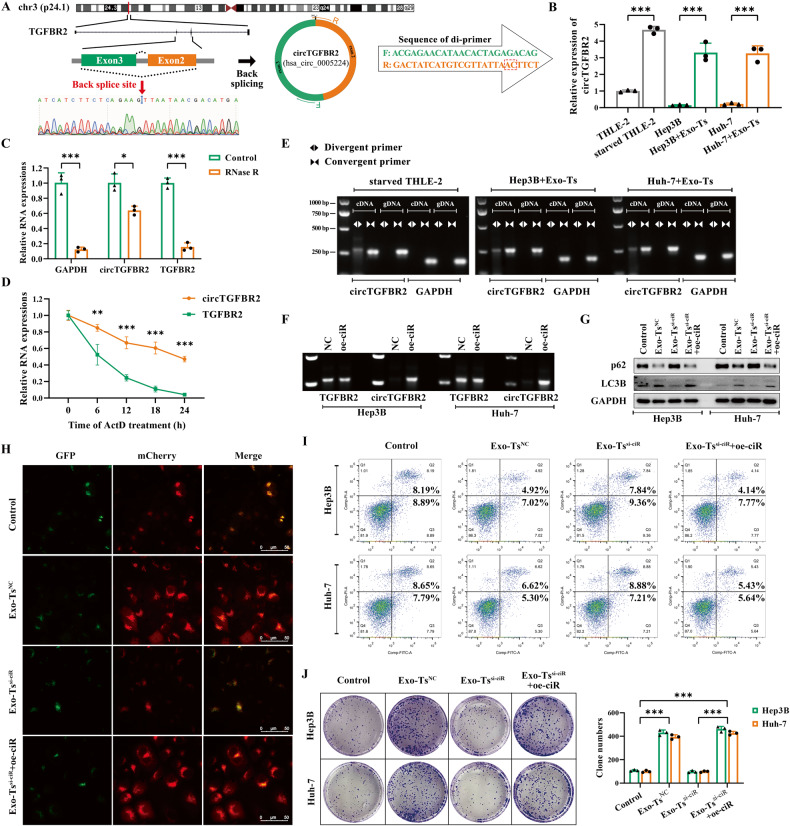
CircTGFBR2 is the effector cargo in Exo-Ts to enhance protective autophagy in HCC cells. **A** Schematic diagram of the back-splicing transcript generated from linear TGFBR2. **B** Relative expression changes of circTGFBR2 in HCC cells cocultured with Exo-Ts were measured by qRT‒PCR assays. **C** RNase R (2.5 U/μg, 37 °C, 15 min) was used to remove the linear transcripts from cellular extracts, leaving circRNAs behind. qRT‒PCR assays were applied to assess the resistance of RNAs to RNase R. **D** RNA decay assay evaluated the stability of circTGFBR2 and TGFBR2 in THLE-2 cells by qRT‒PCR after ActD (1 μg/ml) treatment. **E** Divergent and convergent primers for circTGFBR2 were applied to amplify both cDNA and gDNA; GAPDH was used as a negative control. Agarose gel electrophoresis visualized the products. **F** Overexpression vectors for circTGFBR2 (oe-ciR) containing front and rear circular frames were constructed. PCR assays were present in HCC cell lines and demonstrated that oe-ciR could accurately express circTGFBR2 but not linear TGFBR2. **G** Western blotting analysis of LC3B and p62 in HCC cell lines cocultured with the indicated exosomes alone or cotransfected with oe-ciR. **H** Autophagic flux in Hep3B cells after the indicated treatments was observed under fluorescence microscopy. **I** Apoptosis was assessed by flow cytometry assay in Hep3B and Huh-7 cells cocultured with the indicated exosomes alone or cotransfected with oe-ciR. **J** The proliferation of HCC cells in low serum concentrations and cocultured with the indicated exosomes alone or cotransfected with oe-ciR was assessed by colony formation assays. The data are presented as the mean ± SD of at least three independent experiments. ***P < 0.001.

According to circBase [29], hsa_circ_0005224 (circTGFBR2) is an exonic circRNA ~360 nt in length originating from exon 2 and exon 3 of the TGFBR2 (transforming growth factor-beta receptor type II) gene on chr3:30686238-30691952. Sanger sequencing further validated that the sequence around the junction site was consistent with the results of RNA-seq and the CircInteractome database [30] (Fig. 4A). The qRT‒PCR results showed that the relative expression of circTGFBR2 was significantly upregulated in starvation-stressed THLE-2 cells and much higher than that in HCC cells, while Exo-Ts significantly increased the content of circTGFBR2 in HCC cells (Fig. 4B). RNase R resistance analysis indicated that circTGFBR2 was much more resistant to RNase R than TGFBR2 and GAPDH (Fig. 4C). When ActD was added to THLE-2 cells for the indicated time periods, circTGFBR2 was much more stable than its linear counterpart (Fig. 4D). In addition, both convergent and divergent primers of circTGFBR2 were applied for amplification, and the circTGFBR2 band was observed only in cDNA samples but not in genomic DNA (Fig. 4E). This evidence suggests that circTGFBR2 is a stable circular transcript that can be efficiently delivered to HCC cells by Exo-Ts. Overexpression vectors for circTGFBR2 (oe-ciR) containing front and rear circular frames were constructed to imitate and investigate the function of circTGFBR2 in HCC cells. The results of the PCR assay showed that oe-ciR could efficiently upregulate the expression of circTGFBR2 in Hep3B and Huh-7 cells but not its linear counterpart (Fig. 4F).

To further confirm the effect of circTGFBR2 on HCC cells, we performed western blotting and autophagic flux assays to assess the autophagic status in cells, while flow cytometry assays using annexin V PI/FITC and clonogenic assays were used to evaluate the apoptotic rate and proliferative ability of HCC cells under starvation stress. Western blotting results suggested that overexpression of circTGFBR2 in Hep3B and Huh-7 cells significantly increased LC3 lipidation and decreased p62 accumulation, similar to coculturing with Exo-Ts, indicating activation of autophagy in HCC cells (Fig. 4G). Moreover, autophagic flux assays showed that both oe-ciR and Exo-Ts could increase the number of autophagosomes (yellow puncta) and autolysosomes (red puncta) in target cells, demonstrating an enhancement of autophagic flux (Fig. 4H). Flow cytometry revealed that coculture with Exo-Ts or overexpression of circTGFBR2 in HCC cells could significantly suppress cell apoptosis under starvation stress (Fig. 4I). However, these effects of Exo-Ts were neutralized when the content of circTGFBR2 in Exo-Ts was suppressed using si-ciR. In addition, a clonogenic assay confirmed that circTGFBR2 plays an essential role in Exo-Ts promoting the proliferation of HCC cells at low serum concentrations, and overexpression of circTGFBR2 in HCC cells significantly promoted the proliferation of HCC cells under starvation stress and effectively rescued the effects of circTGFBR2 deficiency in Exo-Ts. (Fig. 4J). All the above results suggested that circTGFBR2 was a significant regulator in Exo-Ts to enhance protective autophagy in HCC cells.

### CircTGFBR2 functions as a sponge for miR-205-5p

**Fig. 5:**
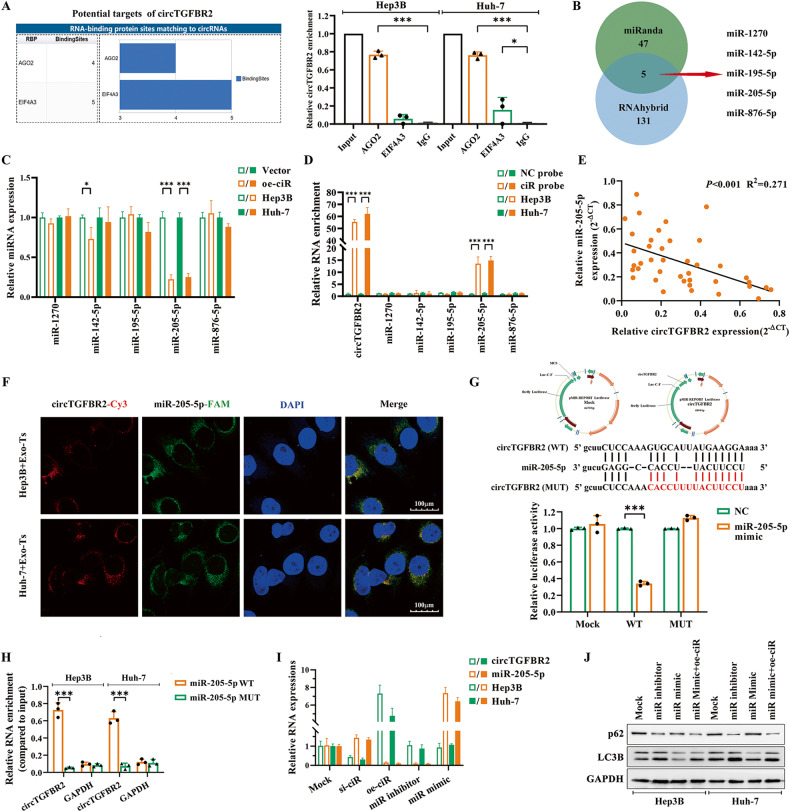
CircTGFBR2 functions as a sponge for miR-205-5p. **A** Prediction analysis of the interaction between circTGFBR2 and RBPs through CircInteractome, and the physical interaction between circTGFBR2 and AGO2 in HCC cells was validated by RIP assay. **B** The Venn diagram shows 5 candidate miRNAs identified from the overlap between the miRanda and RNAhybrid databases. **C** Relative expression changes of the candidate miRNAs in HCC cells transfected with oe-ciR were measured by qRT‒PCR assays. **D** Interaction between circTGFBR2 and the candidate miRNAs was validated by RNA pulldown assay using an antisense probe spanning the junction site of circTGFBR2 (ciR probe). **E** A negative correlation between the expression of circTGFBR2 and miR-205-5p was observed in HCC tumors and peritumoral normal tissues (*P* < 0.001, *R*^2^ = 0.271) **F** RNA-FISH indicates the location of circTGFBR2 and miR-205-5p in HCC cells. CircTGFBR2 was labeled red with Cy3; miR-205-5p was labeled green with FAM; Nuclei were stained blue with DAPI. **G** Dual luciferase reporter assay was used to detect the relative luciferase activity (firefly/Renilla) in 293 T cells cotransfected with miR-205-5p mimics and pMIR-circTGFBR2 WT/MUT. **H** RNA pulldown assays confirmed that the overexpressed circTGFBR2 in HCC cells was dramatically enriched by the biotinylated miR-205-5p. **I** Results of qRT‒PCR assay show the regulatory relationship between circTGFBR2 and miR-205-5p in HCC cells. **J** Western blotting analysis validated the effect of miR-205-5p on autophagy in HCC cells. The data are presented as the mean ± SD of at least three independent experiments. ***P < 0.001, *P < 0.05.

Available studies have demonstrated that circRNAs mainly exert their biological functions by acting as miRNA sponges to bind miRNAs and then promote miRNA-targeted gene expression [16]. According to the CircInteractome database, two RBPs bear multiple binding sites matching circTGFBR2: AGO2 (Argonaute 2) and EIF4A3 (Eukaryotic initiation factor 4A-III). Based on the overexpression of circTGFBR2, RIP assays were performed in Hep3B and Huh-7 cells with antibodies targeting AGO2 and EIF4A3. The results revealed that the relative level of circTGFBR2 in the AGO2-enriched samples was much higher than that in the EIF4A3- or IgG-enriched samples, suggesting that circTGFBR2 may act as a miRNA sponge through the AGO2 protein (Fig. 5A). Thus, we used the miRanda and RNAhybrid databases to predict the potential target miRNAs of circTGFBR2, and 5 candidate miRNAs were identified from the overlap between the databases (Fig. 5B). Then, Hep3B and Huh-7 cells were transfected with oe-ciR, and the relative expression of candidate miRNAs in HCC cells was measured by qRT‒PCR. As shown in Fig. 5C, the expression of miR-205-5p was significantly downregulated by oe-ciR. In addition, an RNA pulldown assay was performed to determine the interaction between circTGFBR2 and the candidate miRNA using an antisense probe spanning the junction site of circTGFBR2 (ciR probe). The results verified that miR-205-5p but not other candidate miRNAs had a significant difference in the pulldown enrichment level by the ciR probe compared to the NC probe (Fig. 5D). The expression of circTGFBR2 and miR-205-5p in HCC tumors and peritumoral normal tissues were negatively correlated by Spearman correlation analysis (*P* < 0.001, *R*^2^ = 0.271) (Fig. 5E). Moreover, FISH assays confirmed the colocalization of circTGFBR2 and miR-205-5p in the cytoplasm, suggesting an interaction between them (Fig. 5F). A circTGFBR2 fragment containing the predicted binding site (wild type or mutant) of miR-205-5p was constructed and inserted downstream of the dual-luciferase reporter gene. Analysis of the dual-luciferase reporter assay indicated that the miR-205-5p mimics significantly reduced the relative luciferase activity of the circTGFBR2-miR-205-5p wild-type group compared with the NC mimic group, while the luciferase activity of the circTGFBR2-miR-205-5p mutant group had no significant change (Fig. 5G). RNA pulldown assays confirmed that the overexpressed circTGFBR2 in HCC cells could be dramatically enriched by the biotinylated miR-205-5p wild-type group rather than its mutant group (Fig. 5H). qRT‒PCR indicated that the content of miR-205-5p in HCC cells exhibited a negative correlation with the expression of circTGFBR2 when this molecule was overexpressed or inhibited, but the content of circTGFBR2 had no significant correlation with the change in expression of miR-205-5p (Fig. 5I). The functional role of miR-205-5p in regulating autophagy in HCC cells was assessed by western blotting, and the results showed that autophagy in HCC cells was activated by the miR-205-5p inhibitor but suppressed by the miR-205-5p mimic. Interestingly, oe-ciR could rescue the autophagic intensity suppressed by miR-205-5p mimic in HCC cells (Fig. 5J). These results suggested that circTGFBR2 delivered by Exo-Ts acted as a sponge of miR-205-5p and abrogated the autophagy-suppressive effect of miR-205-5p in HCC cells.

### Exosomal circTGFBR2 binds miR-205-5p to upregulate the transcriptional activity of ATG5 and enhance the protective autophagy in HCC cells

**Fig. 6:**
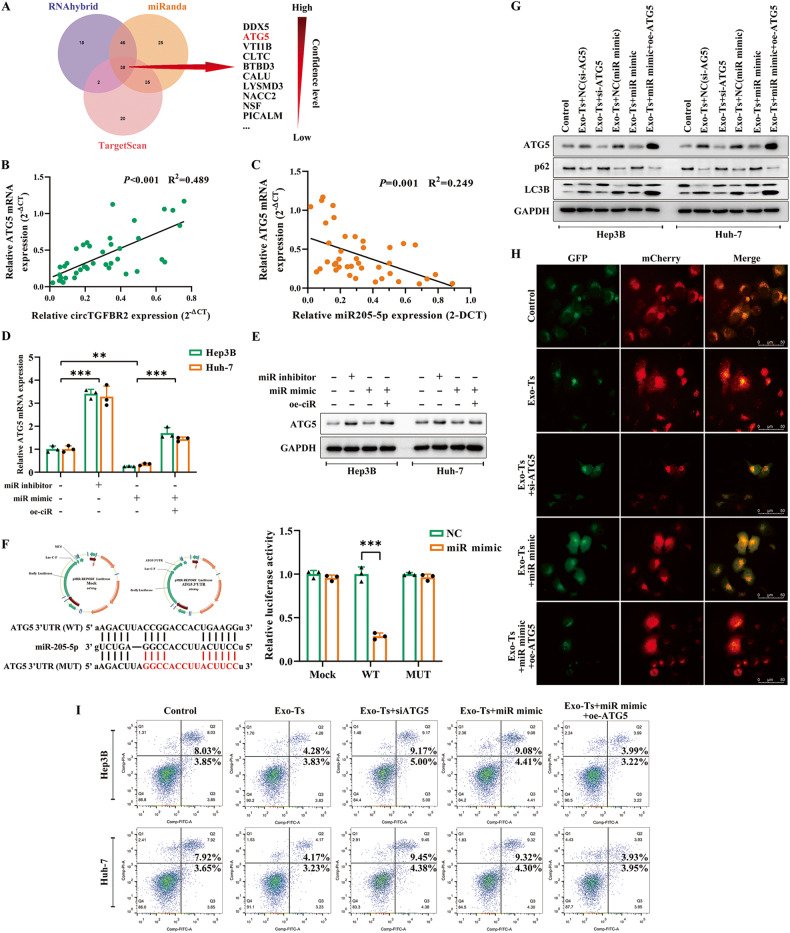
Exosomal circTGFBR2 binds miR-205-5p to upregulate the transcriptional activity of ATG5. **A** Venn diagram showing targets of miR-205-5p predicted from RNAhybrid, miRanda and TargetScan. **B**, **C** A positive correlation between circTGFBR2 and ATG5 mRNA expression (*P* < 0.001, *R*^2^ = 0.489), as well as a negative correlation between miR-205-5p and ATG5 mRNA expression (*P* < 0.001, *R*^2^ = 0.271), was observed in HCC tumors and peritumoral normal tissues (*P* < 0.001, *R*^2^ = 0.249). **D**, **E** The regulation of ATG5 transcription and translation by miR-205-5p in HCC cells was investigated by qRT‒PCR and western blotting assays. **F** Dual luciferase reporter assay was used to detect the relative luciferase activity in 293 T cells cotransfected with miR-205-5p mimics and pMIR-ATG5 3′UTR WT/MUT. **G** Western blotting analysis shows the expression of ATG5 in HCC cells cocultured with Exo-Ts alone or cotransfected with miR-205-5p mimics while showing the rescue effect of ATG5 overexpression vector (oe-ATG5) on the autophagic inhibition of miR-205-5p mimics. **H** Autophagic flux in Hep3B cells after the indicated treatments was observed under fluorescence microscopy. **I** Apoptosis was assessed by flow cytometry assay in Hep3B and Huh-7 cells after the indicated treatments. The data are presented as the mean ± SD of at least three independent experiments. ***P < 0.001, **P < 0.01.

Three databases (RNAhybrid, miRanda, and TargetScan) were used to predict the potential target genes of miR-205-5p in HCC cells, and the autophagy-related ATG5 gene was further examined (Fig. 6A). The relationship between circTGFBR2 and ATG5 mRNA expression or between miR-205-5p and ATG5 mRNA expression in HCC tumors and peritumoral normal tissues was confirmed by Spearman correlation analysis (Fig. 6B, C). The regulation of ATG5 transcription and translation by miR-205-5p in HCC cells was investigated by qRT‒PCR and western blotting assays. The results indicated that the ATG5 mRNA and protein expression in Hep3B and Huh-7 cells was negatively correlated with the content of miR-205-5p in HCC cells, while this downregulation induced by miR-205-5p mimics could be reversed as a result of overexpression of circTGFBR2 (Fig. 6D, E). A dual-luciferase reporter assay was performed to confirm the binding relationship between miR-205-5p and ATG5 mRNA. Firefly/Renilla luciferase vectors bearing the ATG5 3′UTR sequence (wild type or mutant) were introduced into 293 T cells along with miR-205-5p mimics. Analysis of dual-luciferase activities showed that the relative luciferase activity was significantly reduced in the 293 T cells cotransfected with miR-205-5p mimics and ATG5 3’UTR wild-type vector but not mutant vector, indicating that miR-205-5p could directly bind to the ATG5 3’UTR and inhibit its activity (Fig. 6F). To further validate the necessity of ATG5 in protective autophagy mediated by Exo-Ts in HCC cells, we detected the levels of autophagy marker proteins (ATG5, LC3II/I, and p62) in Hep3B and Huh-7 cells by western blotting assays, while the autophagic status in HCC cells was investigated by autophagic flux assays. As shown in Fig. 6G, H, knockdown of ATG5 blocked Exo-Ts-enhanced autophagy in HCC cells, as did miR-205-5p mimics, while overexpression of ATG5 was able to rescue the blocking effect of miR-205-5p mimics on Exo-Ts-enhanced autophagy. Moreover, the results of flow cytometry assays revealed that overexpression of ATG5 could rescue the effect of miR-205-5p mimics in blocking Exo-Ts to enhance the starvation resistance of HCC cells (Fig. 6I). Therefore, these results confirmed that ATG5 is directly targeted by miR-205-5p and indirectly regulated by exosomal circTGFBR2, and the regulated ATG5 accounts for the Exo-Ts-enhanced protective autophagy in HCC cells.

### CircTGFBR2 significantly promoted HCC progression and tumor cell autophagy in vivo

**Fig. 7:**
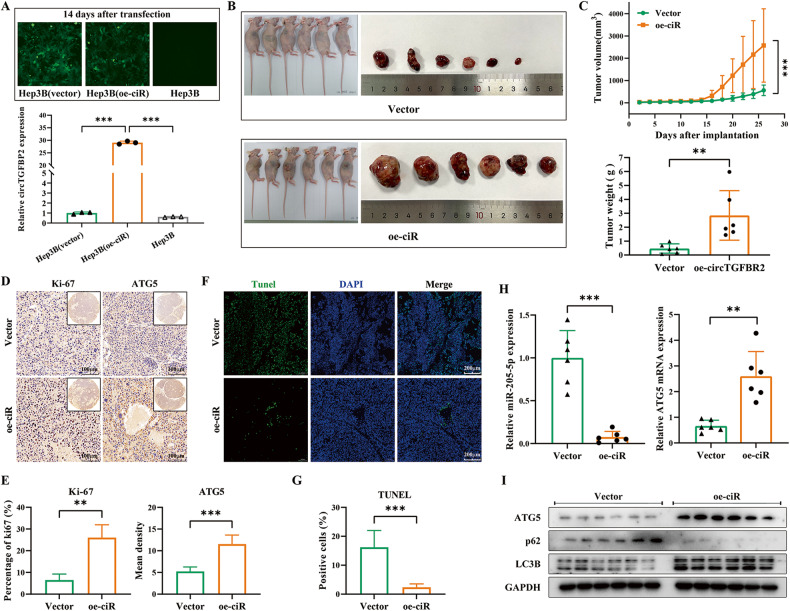
CircTGFBR2 promoted HCC progression and tumor cell autophagy in vivo. **A** Validation of stable circTGFBR2-overexpressing Hep3B cells (Hep3B(oe-ciR)) established by transfecting with pSLenti-EF1-EGFP-F2A-Puro-CMV-S-circTGFBR2-WPRE vectors containing front and rear circular frames. **B**, **C** Xenograft tumors of nude mice 26 days after injection of Hep3B(oe-ciR) or Hep3B(vector) cells (*n* = 6 per group). Tumor growth curves were measured and plotted every 2 days after injection, while tumor weight was measured at the endpoint. **D**, **E** IHC staining was implemented to evaluate the expression of Ki-67 and ATG5 in tumor tissues. The results were quantified using Image-Pro Plus 6.0 and are shown as the percentage of positive cells (Ki-67) or the mean density (ATG5). **F**, **G** TUNEL staining was implemented to evaluate cell apoptosis in tumor tissues. The results were quantified using Image-Pro Plus 6.0 and are shown as the percentage of positive cells. **H** The expression of miR-205-5p and ATG5 mRNA in xenograft tumor tissues was measured by qRT‒PCR. **I** Western blotting analysis of ATG5 and the autophagy-related proteins LC3B and p62 xenograft tumor tissues. The data are presented as the mean ± SD of at least three independent experiments. ***P < 0.001, **P < 0.01.

**Fig. 8:**
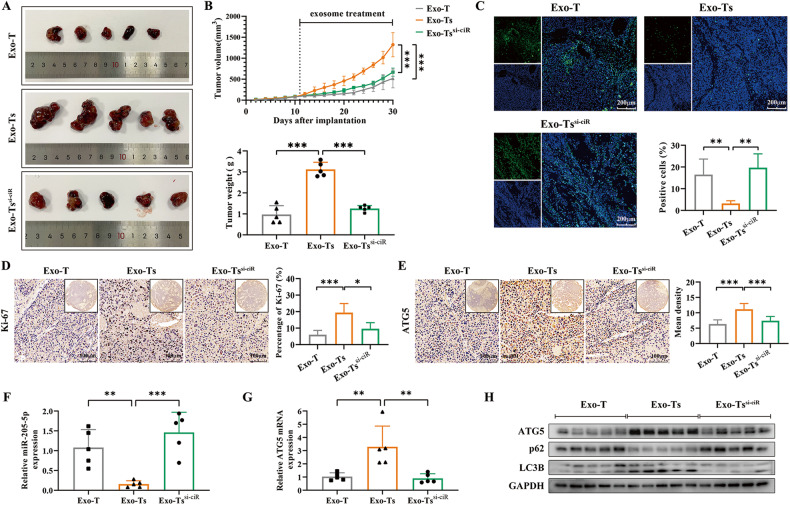
Exosomes divided from starvation-stressed hepatocytes exert a tumor growth-promoting effect in vivo depending on the delivery of circTGFBR2. **A** Xenograft tumors of nude mice 20 days after intraperitoneal injection (i.p) of Exo-T, Exo-Ts or Exo-Ts^si-ciR^ (*n* = 5 per group). **B** Tumor growth curves were measured and plotted every 2 days after injection, while tumor weight was measured at the endpoint. **C** TUNEL staining was implemented to evaluate cell apoptosis in tumor tissues. **D**, **E** IHC staining was implemented to evaluate the expression of Ki-67 and ATG5 in tumor tissues. **F**, **G** The expression of miR-205-5p and ATG5 mRNA in xenograft tumor tissues was measured by qRT‒PCR. **H** Western blotting analysis of ATG5 and the autophagy-related proteins LC3B and p62 xenograft tumor tissues. The data are presented as the mean ± SD of at least three independent experiments. ***P < 0.001, **P < 0.01, *P < 0.05.

To explore the effects of circTGFBR2 in vivo, we first constructed Hep3B (oe-ciR) cell lines with stable overexpression of circTGFBR2 by transfecting lentiviruses into Hep3B cells (Fig. 7A). Then, Hep3B(oe-ciR) cells (oe-ciR group) and Hep3B(vector) cells (vector group, for a negative control) were subcutaneously injected into the right flank of BALB/c nude mice. These mice were monitored closely for tumor growth for 26 days and then sacrificed, and the tumors were weighed. The results showed that tumors derived from Hep3B(oe-ciR) cells were significantly larger than those derived from Hep3B(vector) cells, both in terms of tumor volume and tumor weight (Fig. 7C), suggesting that circTGFBR2 overexpression in HCC cells could promote tumor growth. The expression of Ki-67 (quantified as the percentage of positive cells) and ATG5 (quantified as the mean density) in tumor tissues were evaluated by IHC staining. The results revealed that Ki-67 and ATG5 expression levels were significantly increased in the tumor tissues from oe-ciR group (Fig. 7D, E). Then, TUNEL staining was implemented to evaluate cell apoptosis in tumor tissues, and the results indicated that overexpression of circTGFBR2 in HCC dramatically suppressed cell apoptosis (Fig. 7F, G). Therefore, overexpression of circTGFBR2 could promote HCC progression in vivo. Next, we extracted total RNA from tumor tissues to investigate the content of miR-205-5p and ATG5 mRNA expression by qRT‒PCR. The results demonstrated that the expression of miR-205-5p was significantly downregulated in tissues from the oe-circTGFBR2 group, while ATG5 mRNA expression was significantly upregulated (Fig. 7H). Additionally, we confirmed that ATG5 protein expression and autophagic levels were increased in the oe-ciR group compared to the vector group by performing western blotting analysis of proteins from tumors (Fig. 7I). Next, we performed intraperitoneal injections of exosomes for mice implanted with Hep3B cells in vivo. As seen in Fig. 8A, B, the tumor growth rate and tumor size in the Exo-Ts group significantly exceeded those in the Exo-T group, and this promotion effect was lost when circTGFBR2 was knocked down in Exo-Ts (the Exo-Ts^si-ciR^ group). Meanwhile, TUNEL staining and Ki-67 IHC staining indicated that the survival and proliferation status of tumor tissues in the Exo-Ts group were significantly better than those in the other two groups (Fig. 8C, D), suggesting that Exo-Ts can exert a tumor growth-promoting effect in vivo depending on the delivery of circTGFBR2. Results of qRT‒PCR showed that the miR-205-5p expression was downregulated in tumor tissues from the Exo-Ts group but rescued when circTGFBR2 was knocked down in Exo-Ts. Correspondingly, the content of ATG5 at both mRNA (assessed by qRT‒PCR assay, Fig. 8F, G) and protein levels (assessed by IHC staining and western blotting assay, Fig. 8E, H) were significantly upregulated in tumor tissues following the injection of Exo-Ts, which led to an enhancement of autophagy in tumor tissues. The above results demonstrate a tumor growth-promoting effect in vivo of exosomes divided from starvation-stressed hepatocytes and the circTGFBR2 contained in them.

## Discussion

Emerging studies have revealed that exosomes can promote tumor onset and progression by regulating crosstalk between normal and cancer cells in the tumor microenvironment via their cargo molecules, including small noncoding RNAs, proteins, and lipids [31, 32]. The bilayer lipid membrane of exosomes provides sufficient protection for their contents to enable efficient intercellular communication [15, 33]. In the present study, we focused on the role of exosomes in intercellular communication between hepatocytes and HCC cells. First, we obtained exosomes derived from hepatocytes under starvation stress and characterized them using TEM, NTA, western blotting, and fluorescence microscopy. Then, these exosomes were isolated and cocultured with HCC cells. Interestingly, we found that exosomes derived from starvation-stressed hepatocytes were able to enhance protective autophagy in HCC cells, which contributed to tumor cell resistance to starvation. Using RNA-seq analysis, we confirmed that multiple circRNAs were significantly enriched in exosomes derived from starvation-stressed hepatocytes compared to regular hepatocytes or HCC cells and identified by knockdown validation that circTGFBR2 may be the primary functional molecule in hepatocytic exosomes to enhance autophagy in HCC cells. Overexpression of circTGFBR2 in HCC cells enhanced cellular autophagy, allowing HCC cells to have a lower apoptotic rate and higher proliferation in response to starvation stress from low serum. These results provided us with a novel focus on exosomal cargo in intercellular communication between hepatocytes and HCC cells, and thus, the detailed mechanism of circTGFBR2 regulation of autophagy in HCC cells was further investigated.

There is growing evidence that circRNAs are aberrantly expressed in various cells and exosomes [34, 35]. As a novel class of noncoding RNAs, circRNAs have recently drawn widespread attention, and the contribution of exosomal circRNAs to tumor onset and progression has also attracted much interest. As a class of RNA with stability against exonucleases as its overarching feature, circRNAs are considered to have great potential as therapeutic RNAs. With a 2–5 fold higher stability than linear RNA, circRNA is thought be valuable in cases where the therapeutic agent must be administered less frequently, or in smaller doses, which would also minimize non-specific side effects [36, 37]. However, many circRNAs have not been fully explored in terms of their functions and regulatory mechanisms. Studies have demonstrated that circRNAs frequently act as miRNA sponges by forming a circRNA-miRNA-AGO2 complex to regulate associated genes and thus impact tumor progression [38, 39] and is considered as a promising therapeutic miRNA antagonist [40]. In addition, circRNAs containing intron sequences have the potential to regulate the transcription of their parental genes [41, 42], while circRNAs with internal ribosomal entry sites (IREs) and open reading frames (ORFs) are capable of encoding proteins and performing related functions [43, 44]. CircTGFBR2 (circBase ID: hsa_circ_0005224) is a circular transcript backspliced from the 2nd and 3rd exons of the TGFBR2 gene with a spliced length of 360 nt. According to our results, circTGFBR2 was verified to be capable of binding to AGO2 by RIP assays, confirming the ability to act as a miRNA sponge. Based on bioinformatic analysis, we predicted that circTGFBR2 delivered by exosomes functioned as a competing endogenous RNA (ceRNA) to sponge miR-205-5p in HCC cells. The results of the dual-luciferase reporter assay and RNA pulldown assay in this study confirmed that circTGFBR2 was able to bind competitively to miR-205-5p. Furthermore, circTGFBR2 abolished the endogenous suppressive effect of miR-205-5p on autophagy. Taken together, these findings indicated that the protective autophagy enhanced by exosomes was regulated via the circTGFBR2/miR-205-5p axis in HCC.

Autophagy, as a pervasive and controlled self-digestion program within cells, is able to regulate intracellular environmental homeostasis through lysosomal degradation processes [45]. This process provides an essential means of cellular refreshment and remodeling and serves as a mechanism for cell survival under various stress conditions [46]. Studies have shown that autophagy may facilitate the adaptation to metabolic stress in cancer cells during periods of nutrient limitation by recycling cellular proteins and organelles [47]. This phenomenon is consistent with our finding that HCC cells achieved higher survivability to starvation stress in response after the cellular autophagy was enhanced by circTGFBR2 in exosomes. Autophagy is mediated and executed by a diverse array of autophagy-associated proteins. These proteins assemble into functional complexes that deliver starvation-induced signals to lipids and regulatory proteins to assemble a double-membrane autophagosome that sequesters large amounts of selected cargoes for degradation [48]. Among these proteins, ATG5 is an important component involved in autophagosome formation/extension and interacts with ATG12/ATG16 to play a key role in autophagy [49]. In the present study, we first found that ATG5 is expressed in an opposite pattern to miR-205-5p in HCC tumor tissues and cell lines, and the results of dual-luciferase reporter assays identified that miR-205-5p could inhibit the transcription of ATG5 by binding directly to its 3′UTR. In rescue experiments, circTGFBR2 abolished the endogenous suppressive effect of miR-205-5p on the target ATG5, while overexpression of ATG5 rescued the blockade of exosome-enhanced autophagy by miR-205-5p mimics in HCC cells. In addition, we verified through in vivo experiments that overexpression of circTGFBR2 in HCC could upregulate the expression of ATG5 in tumor tissues, which enhances autophagy in tumor cells and promotes tumor progression. These observations, in combination with our identification of circTGFBR2 as a primary functional molecule in hepatocytic exosomes, indicate that the protective autophagy enhanced by exosomes is regulated via the circTGFBR2/miR-205-5p/ATG5 axis in HCC. These findings suggest that circTGFBR2 may act as a therapeutic RNA in exosomal vectors, exerting a therapeutic effect by attenuating the promotion of HCC from protective autophagy. This could be a potential therapeutic option for HCC patients who have failed anti-vascular therapy or TAE treatment and deserves further investigation.

However, there are still limitations in this study. The lack of studies on the clinical significance of exosomal circTGFBR2 in the serum of HCC patients is one of the major limitations. Furthermore, the clinical significance of exosomal circTGFBR2 in tissues and serum of HCC patients needs to be further investigated in multicenter, larger samples, which would provide a better understanding of the diagnostic and prognostic value of circTGFBR2 and would be important for clinicians to develop anticipative therapeutic strategies.

## Conclusion

In conclusion, our findings demonstrate that exosomes derived from starvation-stressed hepatocytes induce the resistance of HCC cells to starvation stress via the enhancement of protective autophagy. We also report a new mechanism by which hepatocytic exosomes enhance protective autophagy in HCC cells via exosomal circTGFBR2 and the circTGFBR2/miR-205-5p/ATG5 axis, contributing new evidence for the crosstalk between hepatocytes and HCC cells and a potential therapeutic target for HCC treatment.

## Supplementary information

Supplementary Figure 1

Supplementary Figure 2

qRT-PCR primer sequences

Sequences of siRNA against candidate circRNA

Sequences of biotin-labeled RNA probe

Original Data File For Western Blots

Reproducibility checklist

## Data Availability

The datasets used and/or analyzed during the current study are available from the corresponding author upon reasonable request.
