# Supplementary figures and images for: Exosomal circTGFBR2 promotes hepatocellular carcinoma progression via enhancing ATG5 mediated protective autophagy
Source: Cell Death Dis. 2023 Jul 20;14(7):451. doi: 10.1038/s41419-023-05989-5 (PMC10359294; doi:10.1038/s41419-023-05989-5)

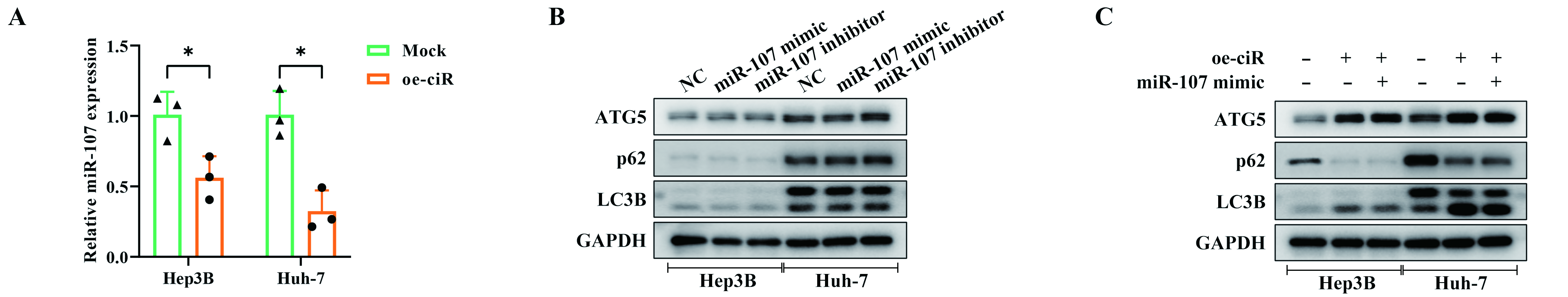

Supplement: Supplementary file 1 — Supplementary Figure 1 [file 41419_2023_5989_MOESM1_ESM.tif]

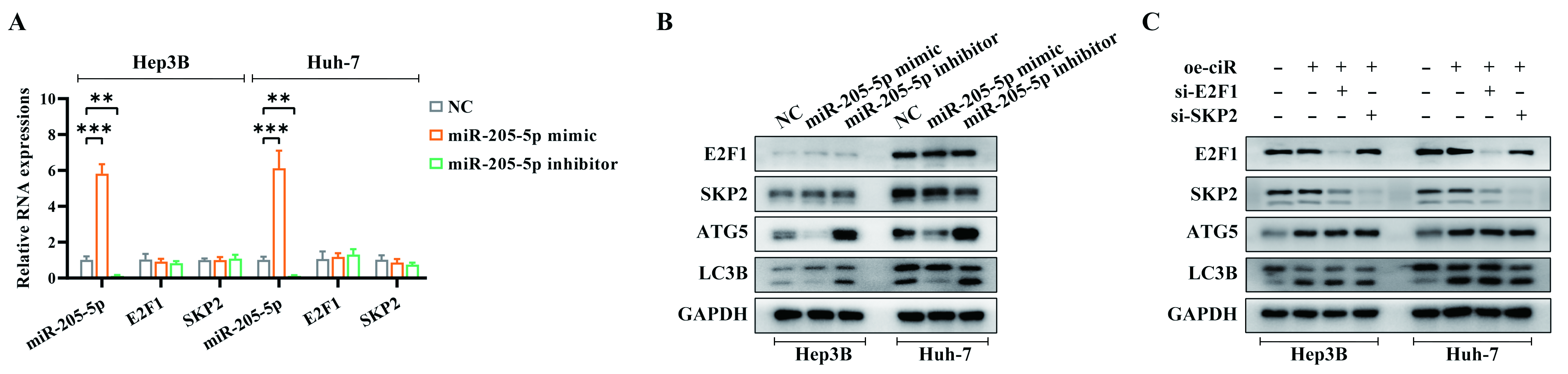

Supplement: Supplementary file 2 — Supplementary Figure 2 [file 41419_2023_5989_MOESM2_ESM.tif]
