# Supplementary material for: Exosomal circTGFBR2 promotes hepatocellular carcinoma progression via enhancing ATG5 mediated protective autophagy
Source: Cell Death Dis. 2023 Jul 20;14(7):451. doi: 10.1038/s41419-023-05989-5 (PMC10359294; doi:10.1038/s41419-023-05989-5)
Supplement: Supplementary file 6 — Original Data File For Western Blots [file 41419_2023_5989_MOESM6_ESM.pdf]

Fig.1A

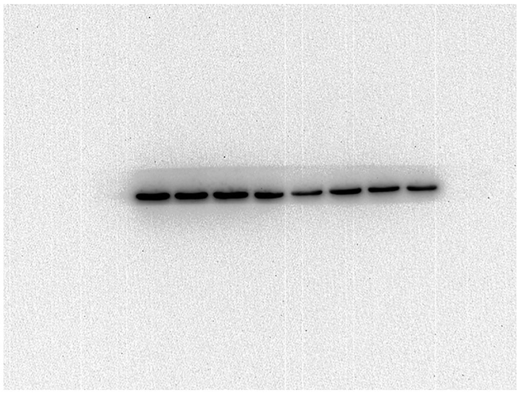

p62

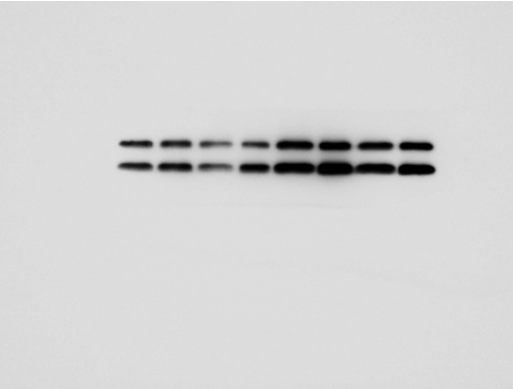

LC3B

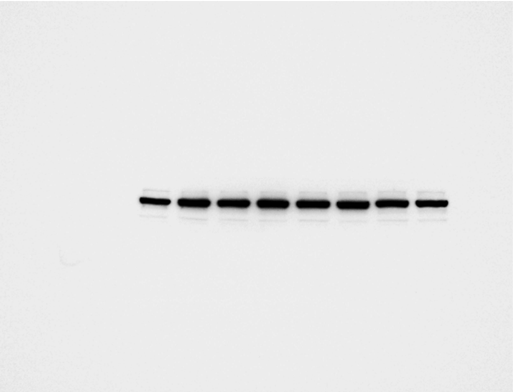

GAPDH

Fig.1D

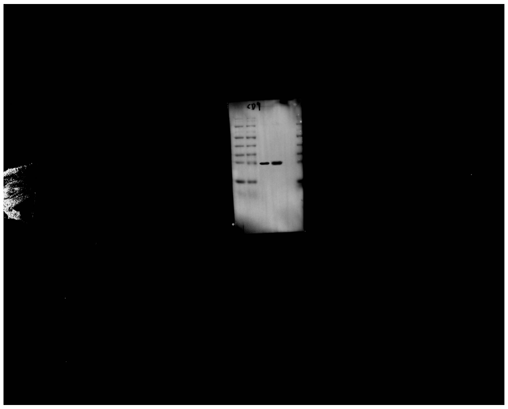

CD9

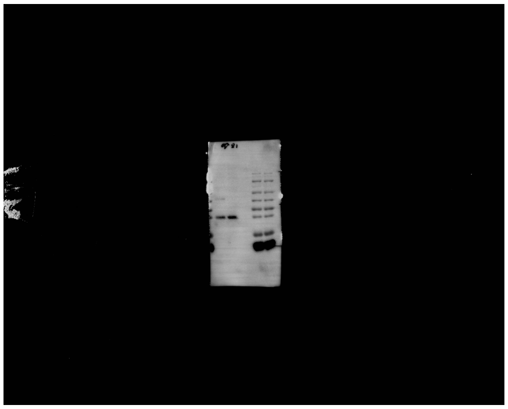

CD81

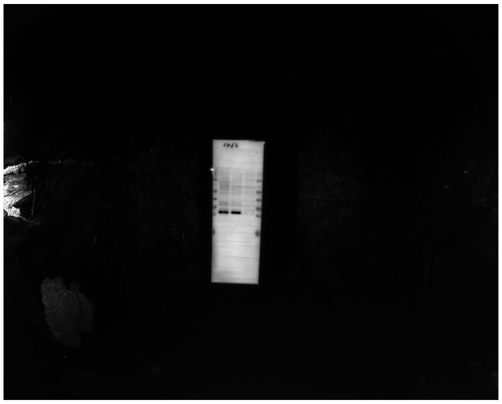

CD63

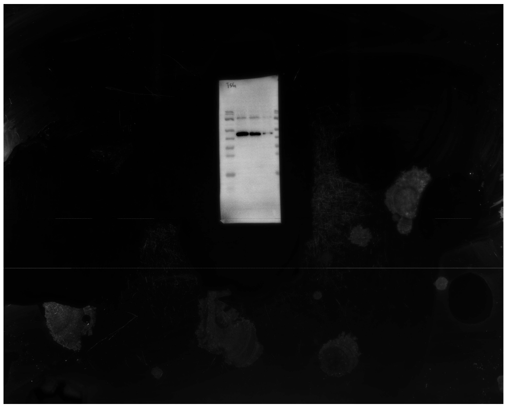

TSG101

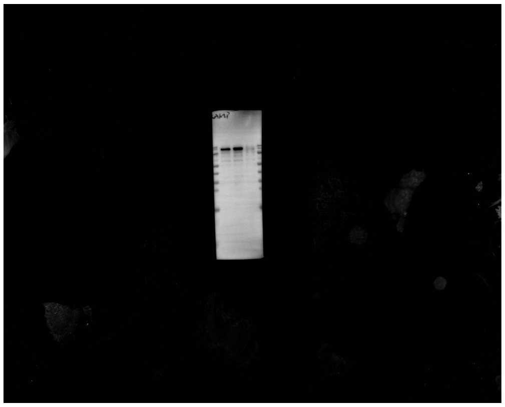

LAMP2

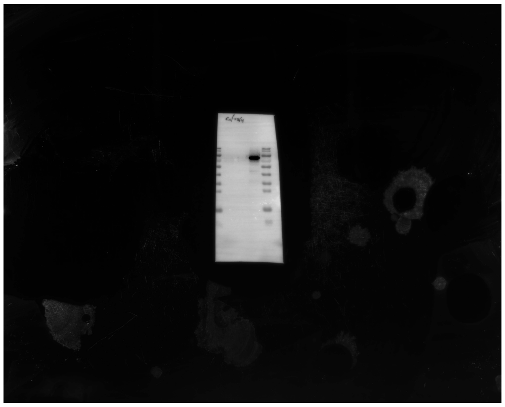

Calnexin

Fig.2A

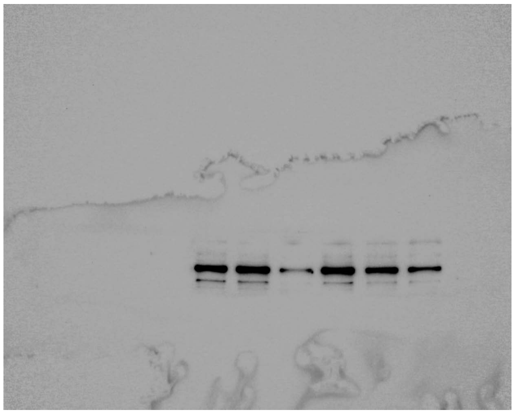

p62

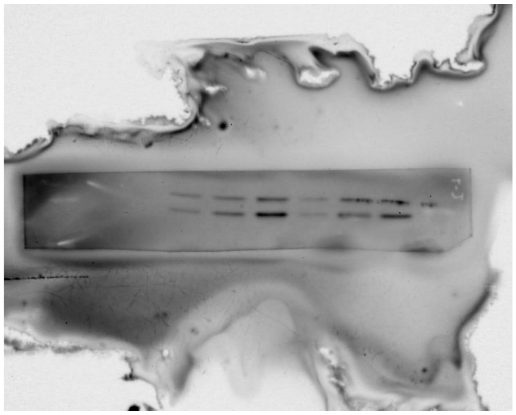

LC3B

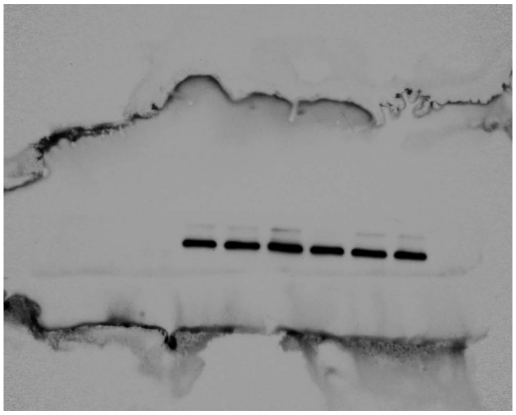

GAPDH

Fig.3G

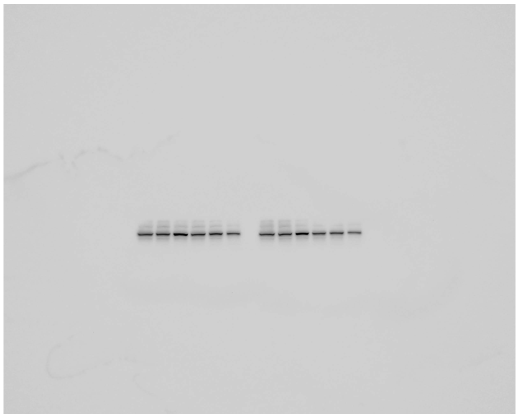

p62

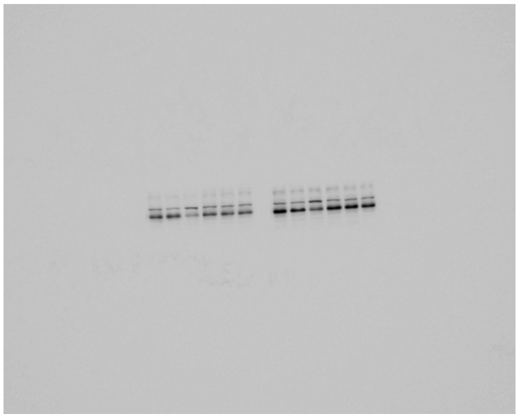

LC3B

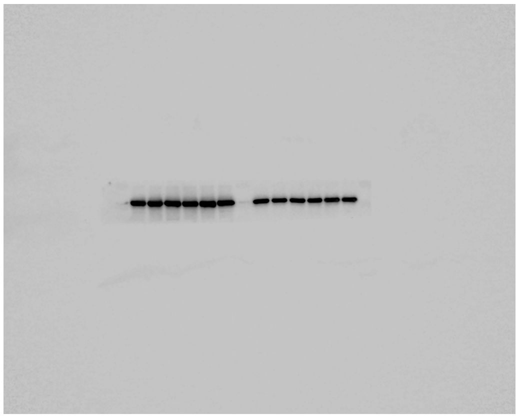

GAPDH

Fig.4G

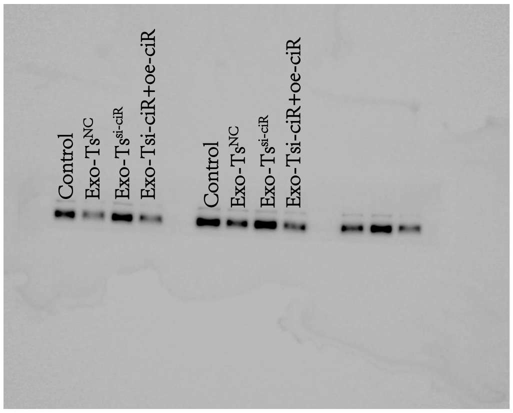

p62

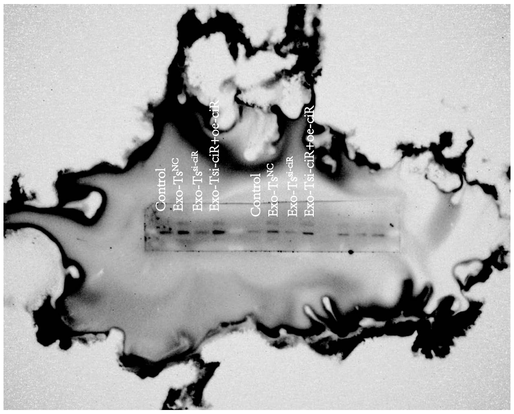

LC3B

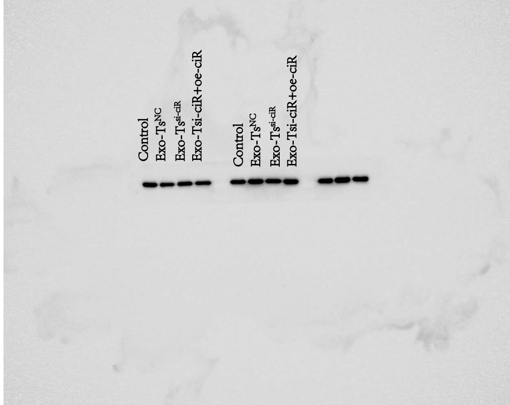

GAPDH

Fig.5J

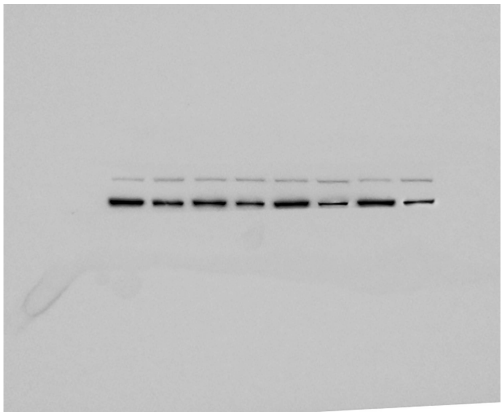

p62

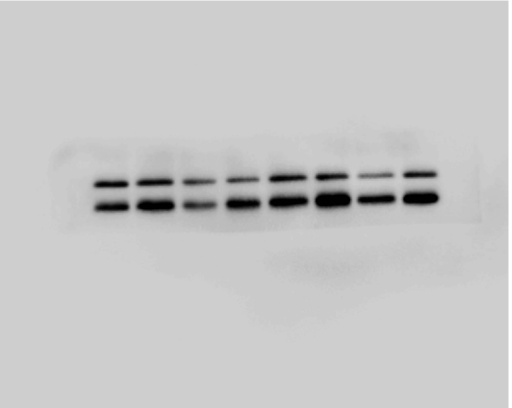

LC3B

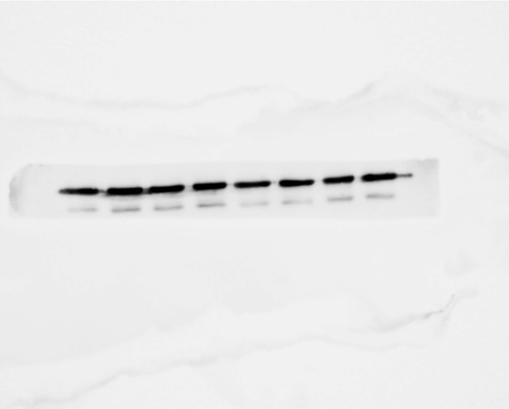

GAPDH

Fig.6E

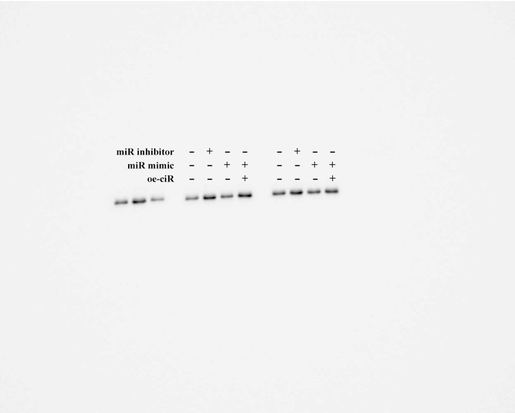

ATG5

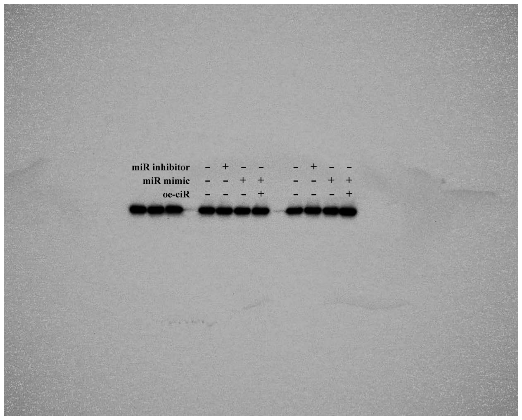

GAPDH

Fig.6G

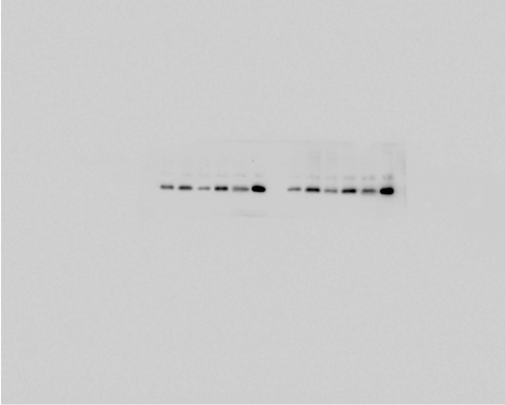

ATG5

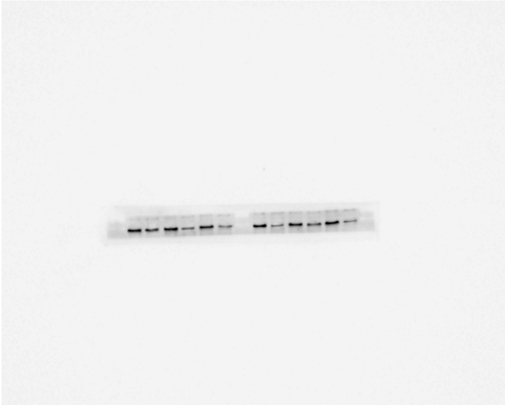

p62

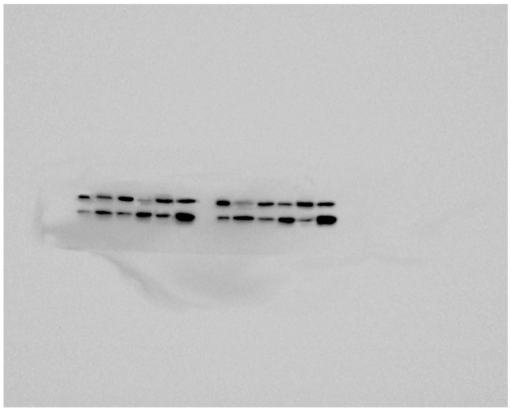

LC3B

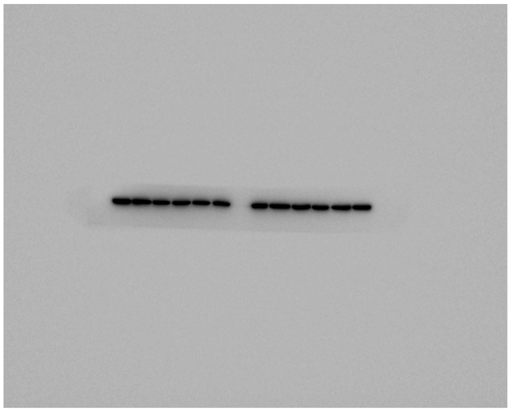

GAPDH

Fig.7I

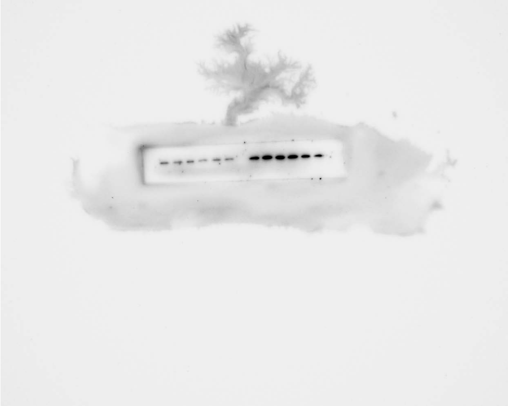

ATG5

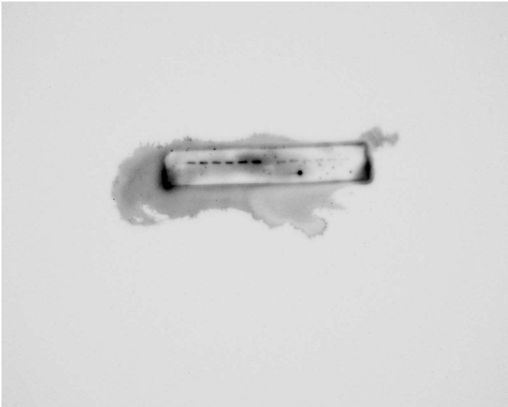

p62

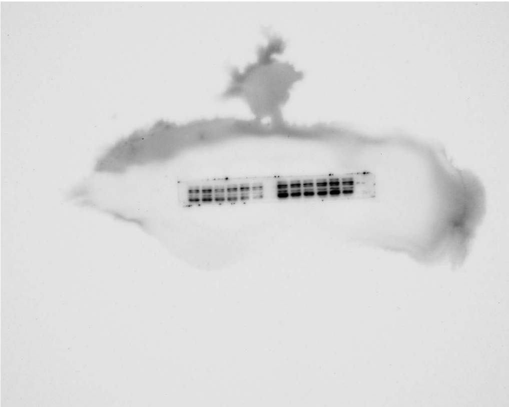

LC3B

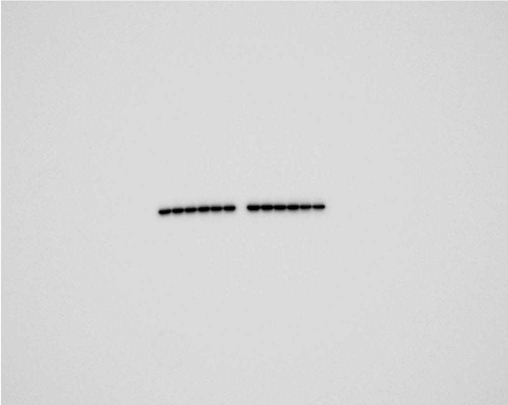

GAPDH

Fig.8H

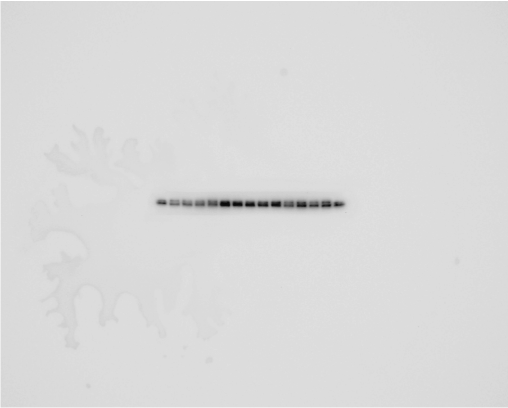

ATG5

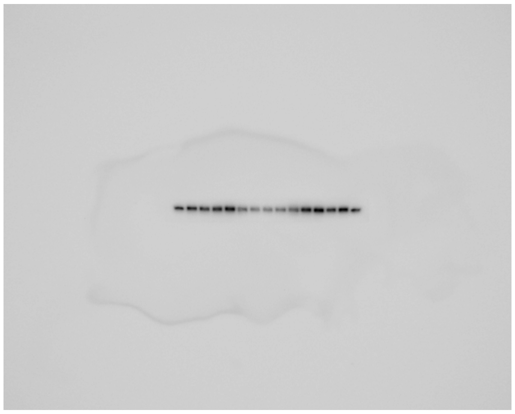

p62

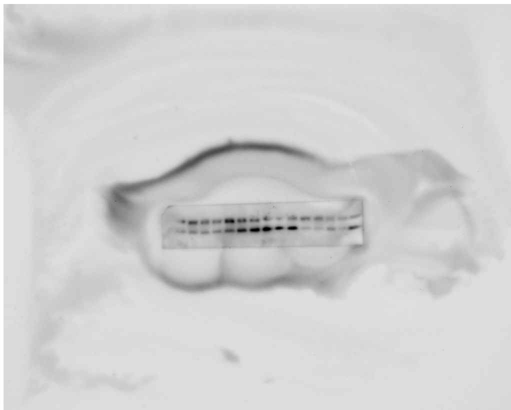

LC3B

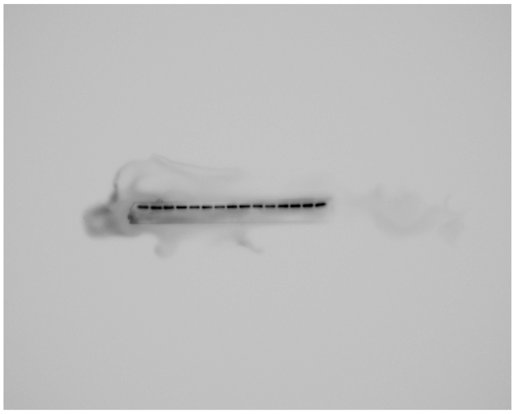

GAPDH

sFig.1B and 1C

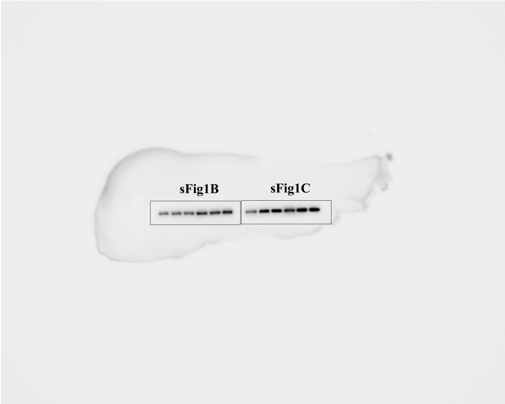

ATG5

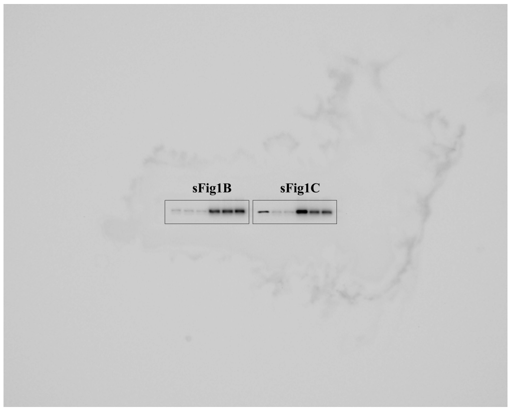

p62

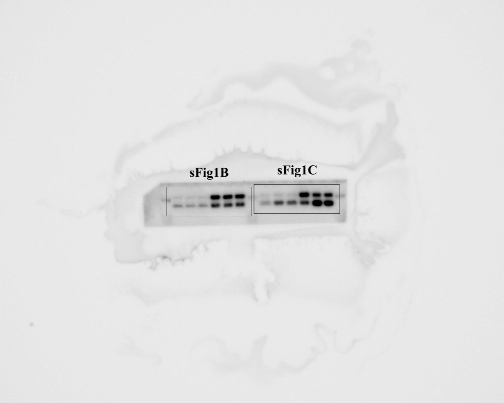

LC3B

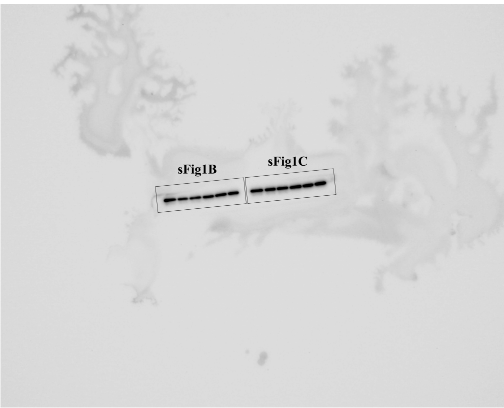

GAPDH

sFig.2B

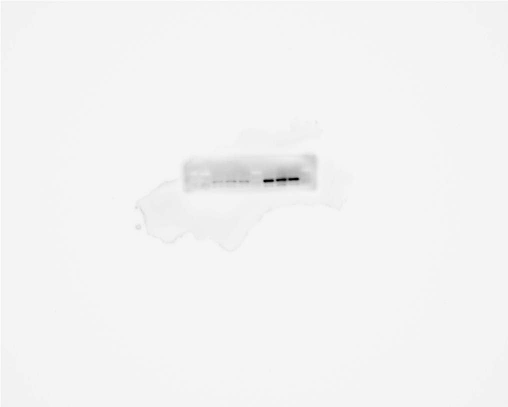

E2F1

SKP2

ATG5

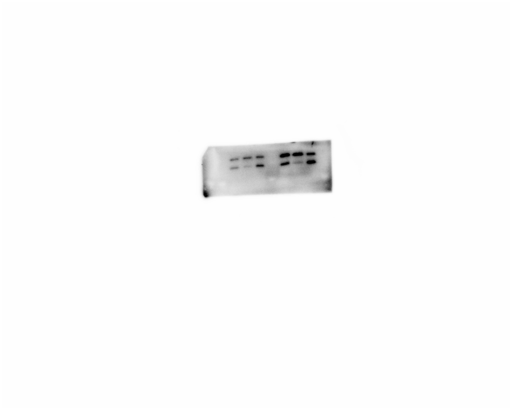

LC3B

GAPDH

sFig.2C

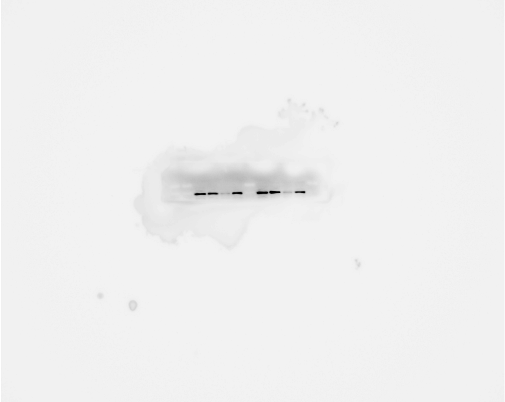

E2F1

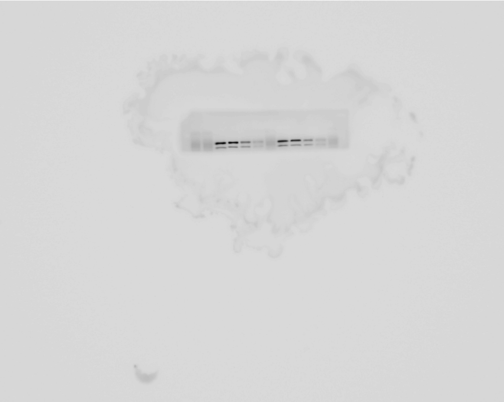

SKP2

ATG5

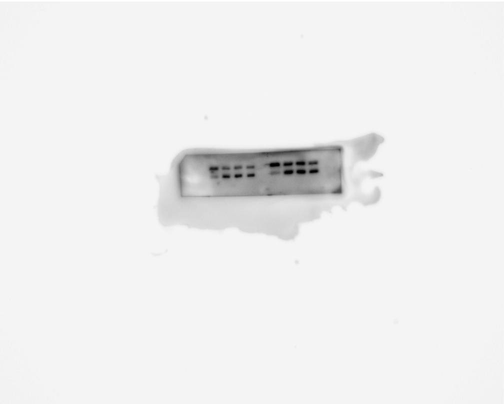

LC3B

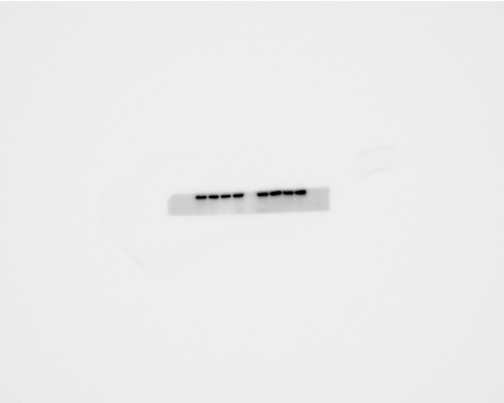

GAPDH
